# Blinded versus unblinded estimation of a correlation coefficient to inform interim design adaptations

**DOI:** 10.1002/bimj.201500233

**Published:** 2016-11-25

**Authors:** Cornelia U. Kunz, Nigel Stallard, Nicholas Parsons, Susan Todd, Tim Friede

**Affiliations:** ^1^ Warwick Medical SchoolUniversity of WarwickGibbet HillCoventryCV4 7ALUK; ^2^ Department of Mathematics and StatisticsUniversity of ReadingWhiteknightsPO Box 220, ReadingRG6 6AXUK; ^3^ Department of Medical StatisticsUniversity Medical Center GoettingenHumboldtallee 32D‐37073 GoettingenGermany

**Keywords:** Blinded, Correlation, Covariance, Estimation, Unblinded

## Abstract

Regulatory authorities require that the sample size of a confirmatory trial is calculated prior to the start of the trial. However, the sample size quite often depends on parameters that might not be known in advance of the study. Misspecification of these parameters can lead to under‐ or overestimation of the sample size. Both situations are unfavourable as the first one decreases the power and the latter one leads to a waste of resources. Hence, designs have been suggested that allow a re‐assessment of the sample size in an ongoing trial. These methods usually focus on estimating the variance. However, for some methods the performance depends not only on the variance but also on the correlation between measurements. We develop and compare different methods for blinded estimation of the correlation coefficient that are less likely to introduce operational bias when the blinding is maintained. Their performance with respect to bias and standard error is compared to the unblinded estimator. We simulated two different settings: one assuming that all group means are the same and one assuming that different groups have different means. Simulation results show that the naïve (one‐sample) estimator is only slightly biased and has a standard error comparable to that of the unblinded estimator. However, if the group means differ, other estimators have better performance depending on the sample size per group and the number of groups.

## 1 Introduction and motivation

The traditional approach to conducting a confirmatory clinical trial is to calculate a fixed sample size in advance of the study. This sample size usually depends on a specified significance level and power but also on other parameters such as variances, mean values, or response rates. While the significance level and power are set by the researcher, the other parameters are usually estimates obtained from previous trials. However, situations occur where these parameters cannot be estimated or can be estimated only with considerable uncertainty at the planning stage of the trial. Designs allowing a re‐assessment of the initial sample size during an ongoing trial have become increasingly popular. Several approaches to estimate the variance in an ongoing trial have been suggested and their performance has been studied. One approach is the common pooled variance estimator that is often used for sample size re‐estimation (see, e.g. Wittes and Brittain, 1990; Birkett and Day, 1994; Coffey and Muller, 1999; Denne and Jennison, 1999; Wittes et al., 1999; Zucker et al., 1999; Kieser and Friede, 2000; Coffey and Muller, 2001; Miller, 2005). This estimator requires unblinding of the treatment group at the time of the interim analysis. As blinding of patients, investigators and the trial team is important in clinical trials to avoid bias (see, e.g. International Conference on Harmonisation of Technical Requirements for Registration of Pharmaceuticals for Human Use (ICH), 1998), regulatory guidelines on adaptive designs encourage the use of blinded methods (European Medicines Agency (EMEA) ‐ Committee for Medicinal Products for Human Use (CHMP), 2007; Food and Drug Administration (FDA), 2010). As a consequence, estimators based on the blinded data set have been proposed. Bristol and Shurzinske (2001) suggested the total variance or one‐sample variance estimator that is unbiased if there are no group differences but otherwise overestimates the within group variance. In order to reduce bias, Gould and Shih (1992) and Zucker et al. (1999) proposed correction methods for the one‐sample variance estimator by subtracting a between‐group variance term from the one‐sample estimator based on an assumed treatment effect. Xing and Ganju (2005) proposed an unbiased estimator based on the blinded data that utilises information about the randomisation block size. They later extended their method to the situation of covariates (Ganju and Xing, 2009). These blinded estimators were recently compared with regard to bias and variance by Friede and Kieser (2013). A review of the various sample size re‐estimation procedures can be found in Friede and Kieser (2006).

In some cases the sample size required can depend on the correlation between different measurements in addition to the variance. However, often there is very little information available on the correlation at the planning stage of a clinical trial. Hence, investigators might want to estimate the correlation in an ongoing trial.

In this paper, we develop and compare estimators for the covariance and the correlation based on blinded data obtained at an interim analysis. The estimators are compared with respect to their bias and their standard error.

In the remainder of this section we provide a brief overview of situations where the value of the correlation is required and it might be beneficial to obtain an estimate of this based on interim data. The rest of our paper is organised as follows: Section 2 introduces the notation used in the paper and describes the different estimators we have considered. Our findings are summarised in Section 3 and we close the paper with the discussion in Section 4.

### 1.1 Multiple primary endpoints

Offen et al. (2007) list some disorders for which regulatory agencies require a treatment to demonstrate a statistically significant effect on multiple endpoints. Tests on each of these endpoints have to be performed at the one‐sided 2.5% significance level before the treatment's effect can be accepted for the particular disorder. The list includes common disorders like migraine but also comprises arthritis, Alzheimer's disease, depression, multiple sclerosis, psoriasis, and rare diseases such as lupus erythrematosus. They also show how the correlation between the primary endpoints can affect the power of a trial in the case of “co‐primary” endpoints, that is situations where statistical significance has to be achieved for all primary endpoints under investigation. For example, even if only two endpoints are considered the overall power decreases from 80% to 64% if the endpoints are independent (using the intersection‐union test approach). This would have to be compensated by a substantial increase in sample size that is a drain on resources and might even be impossible in some settings such as rare diseases. Based on the work of Offen et al., Chuang‐Stein et al. (2007) propose a new method for the same situation using a mixed frequentist and Bayesian approach. Although their method has a smaller sample size than the intersection‐union approach, it still depends on the correlation between the endpoints. Furthermore, Lucadamo et al. (2012) study different solutions for estimating the power of the intersection‐union test. Another situation where the correlation between multiple primary endpoints is of concern is where the trial is deemed positive if a statistically significant result is obtained for at least one of the endpoints under consideration. Li and Mehrotra (2008), for example, develop a method where the significance level for a second primary endpoint depends on the observed *p*‐value for the first one. Their approach also depends on the correlation between the endpoints.

### 1.2 Multiple short‐term surrogate endpoints

Other situations where the correlation between measurements can be of importance for the performance of a method include the use of short‐term (surrogate) secondary endpoints to inform decisions in an ongoing trial. Galbraith and Marschner (2003) discuss interim analyses for clinical trials where an endpoint is observed repeatedly during follow‐up, with the last observation being considered the primary endpoint. They show that the correlation between the measurements taken at different time points can be exploited in order to increase the precision of the estimate of the primary endpoint effect. Todd and Stallard (2005) describe a method for adaptive seamless phase II/III designs where a secondary endpoint is incorporated into the trial design in order to select the most promising treatment at an interim analysis (see also Stallard, 2010; Kunz et al., 2015). Furthermore, Kunz et al. (2014) developed an approach to select the most promising treatment based on interim analysis data incorporating a short‐term endpoint. The last methods depend on the ability to obtain an unbiased estimate of the correlation coefficient in an ongoing trial, often based on very little data.

## 2 Statistical methods

### 2.1 Notation

Let G≥2 denote the number of arms within a multi‐arm, randomised, controlled, double‐blind trial and let $n_{g}$ denote the number of patients in group *g* (g=1⋯G) for which data are available with $n=\sum_{g=1}^{G}n_{g}$. Furthermore, let G(i) denote a function indicating group membership for patient *i*, that is with G(i)=g if patient *i* is in group *g*. Assume that in each of the *G* groups two measurements ($x_{i}$ and $y_{i}$) per patient are taken that follow a bivariate normal distribution with

$$\left(\begin{array}{c} X_{i}\\ Y_{i} \end{array}\right)∼N\left(\left(\begin{array}{c} \mu_{x_{G(i)}}\\ \mu_{y_{G(i)}} \end{array}\right),\left(\begin{array}{cc} \sigma_{x_{G(i)}}^{2} & \rho \sigma_{x_{G(i)}}\sigma_{y_{G(i)}}\\ \rho \sigma_{x_{G(i)}}\sigma_{y_{G(i)}} & \sigma_{y_{G(i)}}^{2} \end{array}\right)\right).$$

Note, that we assume the correlation to be independent of the group, that is the correlation between *X* and *Y* is the same within each group *g*. Furthermore, while $\mu_{x_{g}}$ and $\mu_{y_{g}}$ denote the unknown population parameters of group *g*, for some methods described below it is necessary to specify values for these parameters that are assumed in order to estimate the covariance or correlation. Let ${\tilde{\mu }}_{x_{g}}$ and ${\tilde{\mu }}_{y_{g}}$ denote these assumed values with ${\tilde{\mu }}_{x_{g}}=\mu_{x_{g}}+\delta_{x_{g}}$, ${\tilde{\mu }}_{y_{g}}=\mu_{y_{g}}+\delta_{y_{g}}$, $\mu_{x}=\sum_{g=1}^{G}\frac{n_{g}}{n}\mu_{x_{g}}$, $\mu_{y}=\sum_{g=1}^{G}\frac{n_{g}}{n}\mu_{y_{g}}$, $\delta_{x}=\sum_{g=1}^{G}\frac{n_{g}}{n}\delta_{x_{g}}$, $\delta_{y}=\sum_{g=1}^{G}\frac{n_{g}}{n}\delta_{y_{g}}$, ${\tilde{\mu }}_{x}=\sum_{g=1}^{G}\frac{n_{g}}{n}{\tilde{\mu }}_{x_{g}}$, and ${\tilde{\mu }}_{y}=\sum_{g=1}^{G}\frac{n_{g}}{n}{\tilde{\mu }}_{y_{g}}$.

We mainly focus on block randomisation but also include results for simple randomisation (Altman and Bland, 1999) for one blinded estimator. Let B(i) denote a function indicating block membership for patient *i*, that is with B(i)=b if patient *i* is in group *b*.

### 2.2 Time of the analysis

In the following, we assume that the time of the analysis is fixed so that estimating the correlation is always done after a fixed number of patients, *n*, is enrolled into the trial. In the case of block randomisation, the number of patients per group, $n_{g}$, is also fixed. If simple randomisation is used only the total number of patients, *n*, is fixed while $n_{g}$ can vary.

### 2.3 Estimation of the covariance

Our ultimate aim is to estimate the correlation ρ. However, we start by focusing on estimating the covariance between *X* and *Y*. Table 1 lists the six different estimators we have investigated. In order to calculate the pooled covariance $cov_{\text{pool}}$, we need to unblind the data. We then calculate the covariance within each group and take a “weighted average” across the groups. If we do not unblind the data, four different estimators for the covariance can be defined. The “naïve” estimator cov_naïve_ is obtained by calculating the covariance for the whole data set, that is treating the data as if they were obtained from just one group. This method can always be applied, hence, we present results not only for block randomisation but also for simple randomisation ($cov_{\text{sr}}$).

**Table 1:** Proposed estimators for the covariance

**Block randomisation**	
Unblinded	
Pooled	$cov_{\text{pool}}=\frac{1}{n}\sum_{g=1}^{G}\frac{n_{g}}{n_{g}-1}\left(\sum_{i:G(i)=g}\left(x_{i}-{\bar{x}}_{g}\right)\left(y_{i}-{\bar{y}}_{g}\right)\right)$
Blinded	
Naïve	$cov_{\text{naïve}}=\frac{1}{n-1}\sum_{i=1}^{n}\left(x_{i}-\bar{x}\right)\left(y_{i}-\bar{y}\right)$
Based on Xing and Ganju	$cov_{\text{XG}}=\frac{B}{n(B-1)}\sum_{b=1}^{B}\left(\sum_{i:B(i)=b}\left(x_{i}-\bar{x}\right)\right)\left(\sum_{i:B(i)=b}\left(y_{i}-\bar{y}\right)\right)$
Based on Zucker et al.	
Using $\bar{x},\bar{y}$	$cov_{\text{Z1}}=\frac{n-1}{n}cov_{\text{naïve}}-\sum_{g=1}^{G}\frac{n_{g}}{n}{\tilde{\mu }}_{x_{g}}{\tilde{\mu }}_{y_{g}}+\bar{x}\bar{y}$
Using ${\tilde{\mu }}_{x},{\tilde{\mu }}_{y}$	$cov_{\text{Z2}}=cov_{\text{naïve}}-\sum_{g=1}^{G}\frac{n_{g}}{n-1}{\tilde{\mu }}_{x_{g}}{\tilde{\mu }}_{y_{g}}+\frac{n}{n-1}{\tilde{\mu }}_{x}{\tilde{\mu }}_{y}$
**Simple randomisation**	
Blinded	$cov_{\text{sr}}=\frac{1}{n-1}\sum_{i=1}^{n}\left(x_{i}-\bar{x}\right)\left(y_{i}-\bar{y}\right)$

The other estimators all require block randomisation. Xing and Ganju (2005) developed an estimator for the variance of an endpoint in an ongoing blinded trial. Their estimator uses the enrollment order of subjects and the randomisation block size to estimate the variance. We extend their method to allow for estimation of the covariance ($cov_{\text{XG}}$) and different sample sizes, variances, and correlations within each group. Zucker et al. (1999) also propose an estimator for the variance based on blinded data. Their estimator incorporates assumptions about the differences in the means between the different groups. Again, we extend their method to allow for estimation of the covariance and more than two groups as well as different variances, sample sizes, and correlations within each group. We present results for two different versions of this estimator: the first one ($cov_{\text{Z1}}$) is based on the estimated overall means for the two endpoints $\bar{x}$ and $\bar{y}$. The second one ($cov_{\text{Z2}}$) is based on the assumed overall means for the two endpoints ${\tilde{\mu }}_{x}$ and ${\tilde{\mu }}_{y}$.

## 3 Results

### 3.1 Analytical expressions for the expected values of estimators for the covariance

For the covariance, analytical expressions for the expected values of the estimators can be obtained. Table 2 shows the expected values for the general case, allowing for different sample sizes, variances, correlations, and means within each group.

**Table 2:** Expected values of the estimators for the covariance

**Block randomisation**	
Unblinded	
Pooled	$E\left[cov_{\text{pool}}\right]=\sum_{g=1}^{G}\frac{n_{g}}{n}\rho \sigma_{x_{g}}\sigma_{y_{g}}$
Blinded	
Naïve	$E\left[cov_{\text{naïve}}\right]=\sum_{g=1}^{G}\frac{n_{g}}{n}\rho \sigma_{x_{g}}\sigma_{y_{g}}+\frac{n}{n-1}\left(\sum_{g=1}^{G}\frac{n_{g}}{n}\mu_{x_{g}}\mu_{y_{g}}-\mu_{x}\mu_{y}\right)$
Based on Xing and Ganju	$E\left[cov_{\text{XG}}\right]=\sum_{g=1}^{G}\frac{n_{g}}{n}\rho \sigma_{x_{g}}\sigma_{y_{g}}$
Based on Zucker et al.	
Using $\bar{x},\bar{y}$	$E\left[cov_{\text{Z1}}\right]=\sum_{g=1}^{G}\frac{n_{g}}{n}\rho \sigma_{x_{g}}\sigma_{y_{g}}-\sum_{g=1}^{G}\frac{n_{g}}{n}\left(\mu_{x_{g}}\delta_{y_{g}}+\mu_{y_{g}}\delta_{x_{g}}+\delta_{x_{g}}\delta_{y_{g}}\right)$
Using ${\tilde{\mu }}_{x},{\tilde{\mu }}_{y}$	$E\left[cov_{\text{Z2}}\right]=\sum_{g=1}^{G}\frac{n_{g}}{n}\rho \sigma_{x_{g}}\sigma_{y_{g}}-\sum_{g=1}^{G}\frac{n_{g}}{n-1}\left(\mu_{x_{g}}\delta_{y_{g}}+\mu_{y_{g}}\delta_{x_{g}}+\delta_{x_{g}}\delta_{y_{g}}\right)$
	$+\frac{n}{n-1}\left(\mu_{x}\delta_{y}+\mu_{y}\delta_{x}+\delta_{x}\delta_{y}\right)$
**Simple randomisation**	
Blinded	$E\left[cov_{\text{sr}}\right]=\sum_{g=1}^{G}\frac{n_{g}}{n}\rho \sigma_{x_{g}}\sigma_{y_{g}}+\sum_{g=1}^{G}\frac{n_{g}}{n}\mu_{x_{g}}\mu_{y_{g}}-\mu_{x}\mu_{y}$

If we assume equal variances $\sigma_{x_{g}}^{2}$ and $\sigma_{y_{g}}^{2}$ within each group *g*, we see that only two estimators for the covariance are unbiased: the pooled estimator $cov_{\text{pool}}$ (based on the unblinded data) and the estimator based on Xing and Ganju ($cov_{\text{XG}}$) as the expected value for both simplifies to $\rho \sigma_{x}\sigma_{y}$ with $\sigma_{x}=\sigma_{x_{g}}$ and $\sigma_{y}=\sigma_{y_{g}}$ for g=1⋯G. With equal variances, the naïve estimators $cov_{\text{naïve}}$ and $cov_{\text{sr}}$ are unbiased if $\mu_{x_{g}}$ and $\mu_{y_{g}}$ are 0 for g=1⋯G. The estimators based on Zucker et al. are both unbiased if $\delta_{x_{g}}=0$ and $\delta_{y_{g}}=0$ for all g=1⋯G. However, the second estimator $cov_{\text{Z2}}$ is also unbiased if a much weaker condition is fulfilled, that is as long as $\delta_{x_{g}}=\delta_{x}$ and $\delta_{y_{g}}=\delta_{y}$ for g=1⋯G holds true.

### 3.2 Application to real data example

Wilcock et al. (2000) report the outcome of a randomised controlled trial of galantamine in patients with mild to moderate Alzheimer's disease. Two different dose levels (24 and 32 mg) were tested against placebo. The primary endpoint was the score on the 11 item cognitive subscale of the Alzheimer's disease assessment scale measured after 6 months. Wilkinson et al. (2001) also report the outcome of a randomised trial of galantamine in patients with Alzheimer's disease. However, they compared three dose levels (18, 24 and 36 mg) to placebo. They used the same primary endpoint but measured after 12 weeks. Wilkinson et al. also report that an interim analysis was carried out after approximately 20 patients per group had completed assessment.

So, in total, there were four treatment groups (dose levels 18, 24, 32 and 36 mg) and the placebo group and two different outcome measures. In such a situation, we might want to use an adaptive seamless Phases II/III design with treatment selection at interim as described by, for example, Todd and Stallard (2005). However, as the method depends on the correlation between the endpoints (see Kunz et al., 2015), we might want to estimate the correlation within the ongoing trial.

Based on Wilcock et al. (2000) and Wilkinson et al. (2001), we simulated data for up to five groups. For the 6‐months endpoint, we used means of 27.1, 25.0, 24.7, 24.5 and 24.4. For the 12‐weeks endpoint, we used means of 29.2, 25.2, 24.8, 24.2 and 23.9. The standard deviation was set to 10 for all groups. The sample size per group was set to either 6 or 24 and the correlation between the endpoint varied between −0.9 and +0.9 in steps of 0.1. For $\delta_{x}$ and $\delta_{y}$ we used the values as shown in Table 3 under Example 2.

**Table 3:** Parameter settings for the simulation study

	Example 1						Example 2					
*g*	$\mu_{x}$	$\mu_{y}$	$\delta_{x}$	$\delta_{y}$	$\sigma_{x}$	$\sigma_{y}$	$\mu_{x}$	$\mu_{y}$	$\delta_{x}$	$\delta_{y}$	$\sigma_{x}$	$\sigma_{y}$
1	0	0	0.1	0.5	1	1	0	0	0.1	0	1	1
2	0	0	0.1	0.5	1	1	0.25	0.25	0.1	−0.125	1	1
3	0	0	0.1	0.5	1	1	0.5	0.5	0.1	−0.25	1	1
4	0	0	0.1	0.5	1	1	0.75	0.75	0.1	−0.375	1	1
5	0	0	0.1	0.5	1	1	1	1	0.1	−0.5	1	1
ρ	−0.8, 0, +0.8											
*G*	2, 3, 5											
$n_{g}$	6, 24											
*B*	2, 3, 6 (if $n_{g}$=6) and 2, 3, 4, 6, 8, 12, 24 (if $n_{g}$=24)											

**Figure 1:**
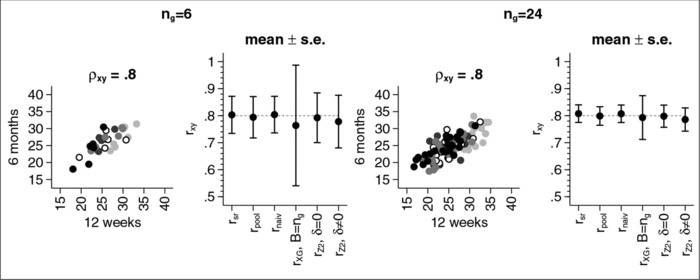
Mean (± s.e.) for the estimate of the correlation coefficient.

Figure 1 shows the results for the real data example. The two scatter plots show examples for how the data might look at the time of the interim analysis. Different markers are used for the different groups. Note that due to the relatively large standard deviation (compared to the relatively small difference between the means) without the different markers, we would not be able to distinguish between the different groups.

For a sample size of $n_{g}=6$, we see that all estimators yield a correlation estimate of about 0.8 except for the estimator based on Xing and Ganju that yields an estimate of 0.76. The latter also has the largest standard error (s.e. 0.22) while the other estimators have a standard error between 0.07 and 0.10.

For a sample size of $n_{g}=24$, the bias of the estimator based on Xing and Ganju gets smaller as does the standard error. However, the standard error for this estimator is still larger than for the other estimators we investigated.

### 3.3 Simulation‐based estimates for the expected values of estimators for the correlation coefficient ρ

In order to obtain a better overview of the properties of the estimators, we simulated data for two examples with different parameter settings that are given in Table 3. For the first example we assumed that all groups have the same means $\mu_{x_{g}}$ and $\mu_{y_{g}}$, which, without loss of generality, were both set to 0. This setting reflects a scenario where the null hypothesis would be true. For the estimators based on the work of Zucker et al. we assumed that $\delta_{x}=0.1$ and $\delta_{y}=0.5$ for all groups. For the second example, we assumed different means and different $\delta_{y}$ for different groups. For both examples, data were simulated for three different values of ρ, for three different values of *G* and for two different values of $n_{g}$. All variances are taken to be equal to 1. For the estimator based on Xing and Ganju, we also considered different values for *B* depending on the sample size $n_{g}$. For each scenario considered we simulated data between 10,000 and 3,000,000 times (depending on how stable the results for the standard error were).

For each simulated dataset, we estimated the covariance and the variances using one of the methods described above. Note that the variances are a special case of the covariance , that is in general VAR[X]=cov[X,X]. Hence, the variances can be obtained using the estimators in Table 1 replacing either *y* with *x* (to obtain the variance of *X*) or *x* with *y* (to obtain the variance of *Y*). We then calculated the correlation *r* using $r=\hat{\rho }=\hat{cov}/\left({\hat{\sigma }}_{x}{\hat{\sigma }}_{y}\right)$.

**Figure 2:**
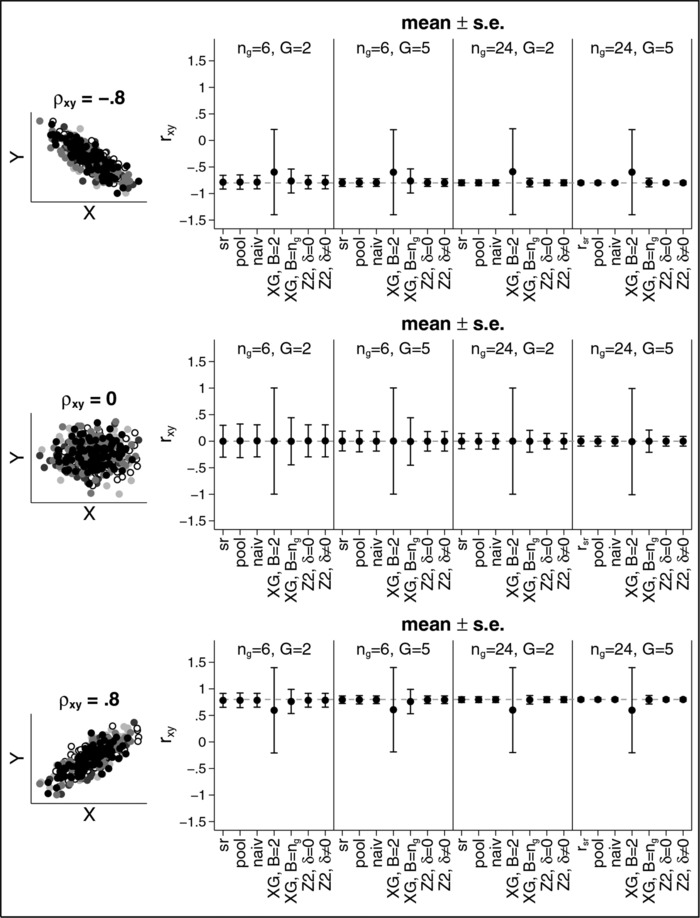
Mean (± s.e.) for the estimate of the correlation coefficient for Example 1.

Figure 2 shows the results for Example 1. Results for $r_{\text{Z1}}$ were omitted in the figure as they were often highly biased. They can still be found in Table A.1 in the Appendix. In the following we will only discuss the results for the other estimators.

The left‐hand side of the figure shows schematic examples of the scatter plots for the scenarios considered. The right‐hand side shows the results for the correlation estimators for different sample sizes $n_{g}$ and different numbers of groups *G*. The upper panel shows the results for ρ=−0.8, the middle panel shows the results for ρ=0 and the bottom panel shows the results for ρ=+0.8. For each method we present the mean and the standard error (s.e.). Overall, nearly all estimators are unbiased except for the estimator based on Xing and Ganju. For a correlation of ρ=±0.8, we obtain $r_{\text{XG}}=\pm 0.6$ if B=2. The expected value of the estimator is not affected by the number of groups *G* nor by the sample size $n_{g}$. The only parameter that affects the results for this particular estimator is the number of blocks *B*. If $B=n_{g}$, the estimator is less biased and has a smaller standard error that can be seen by comparing the results for $n_{g}=6$ with $n_{g}=24$. If ρ=±0.8, $n_{g}=6$ and $B=n_{g}=6$, the estimated correlation is $r_{\text{XG}}=\pm 0.76\left(s.e.\pm 0.23\right)$ while if $n_{g}=24$ and $B=n_{g}=24$, the estimated correlation is $r_{\text{XG}}=0.79\left(s.e.\pm 0.08\right)$. If ρ=0, the estimator based on Xing and Ganju is unbiased but still has the largest standard error irrespective of the sample sizes, the number of groups or the number of blocks.

All other estimators lead to very similar results. In all cases the estimators are either unbiased or the bias is very small compared to the standard error. The standard errors depend on the sample sizes and the number of groups, with larger sample sizes and more groups leading to smaller standard errors. It might be noteworthy that the standard error also depends on the correlation ρ, with ρ=0 leading to the largest standard error for all estimators.

**Figure 3:**
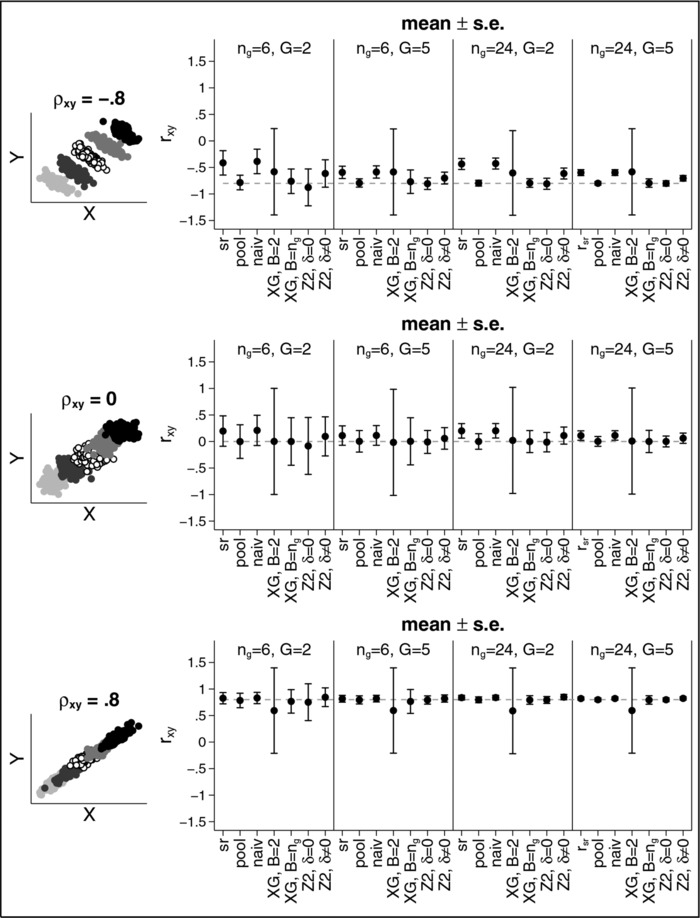
Mean (± s.e.) for the estimate of the correlation coefficient for Example 2.

The situation changes if the group means are different as can be seen from Figure 3 (again results for $r_{\text{Z1}}$ are omitted from the figure, but are included in the Appendix in Table A.2). Now, nearly all estimators are biased except for the “pooled” one which requires unblinding. Largest bias occurs for the naïve estimator for ρ=−0.8, $n_{g}=6$ and G=2. In this case, the average estimated correlation is $r_{\text{naïve}}=-0.41$ with a standard error of 0.23. While the bias gets smaller if the number of groups increases, the sample size per group does not have much impact. For example, for ρ=−0.8 the estimated correlation is −0.41 (−0.43) for G=2 and $n_{g}=6$ ($n_{g}=24$), −0.53 (−0.54) for G=3 and $n_{g}=6$ ($n_{g}=24$) and −0.59 (−0.60) for G=5 and $n_{g}=6$ ($n_{g}=24$). However, the standard error clearly decreases for larger sample sizes and more groups. For example, for ρ=−0.8 the standard error is 0.23 for $n_{g}=6$ and G=2, but only 0.05 for $n_{g}=24$ and G=5.

The estimator based on the work of Xing and Ganju is less biased than the naïve estimator. However, especially if data for only two blocks is available, the standard error is very large. For ρ=±0.8 we obtain a standard error of about 0.80 and for ρ=0 we get 1. Hence, if we calculate a 95% confidence interval it would actually span the entire range of possible values for ρ from −1 to 1. Results for this estimator improve when $B=n_{g}$. However, large standard errors can still occur.

The estimator based on the work by Zucker et al. is also biased, even if $\delta_{x}$ and $\delta_{y}$ are 0, that is, even if we guess the true population means correctly, we still under‐ or overestimate the correlation. For example, for $n_{g}=6$ and G=2, we get $r_{\text{Z2}}=-0.88$ (for ρ=−0.8), $r_{\text{Z2}}=-0.08$ (for ρ=0), and $r_{\text{Z2}}=0.76$ (for ρ=+0.8). While results improve for larger sample sizes and more groups, the estimator still depends on $\delta_{x}$ and $\delta_{y}$, that is on the ability to correctly “guess” the differences between the group means. It also should be noted that for $n_{6}=6$ and G=2, the standard error of the estimators based on the work of Zucker at al. can be quite substantial and sometimes even larger than the one for the estimator based on Xing and Ganju for B=2. Further results for other scenarios can be found in the online Supporting Information.

## 4 Discussion

In this paper, we have considered a number of estimators for the correlation coefficient based on blinded and unblinded data to inform interim decisions in flexible designs and have compared their performance. We have mainly focused on block randomisation and blinded estimators.

Unsurprisingly, the unblinded estimator is only slightly biased and tends to have the smallest standard error in all investigated settings. However, it requires unblinding, whereas maintaining the blind is considered to be one of the most important techniques (together with randomisation) to eliminate or minimise bias (International Conference on Harmonisation of Technical Requirements for Registration of Pharmaceuticals for Human Use (ICH), 1998).

Under the null hypothesis, the naïve estimator performs best out of all estimators based on blinded data. Furthermore, it requires no assumptions and no information other than the data for the two variables under consideration. Yet, its performance is similar to the unblinded estimator for this scenario. However, under the alternative, the bias of this estimator can be substantial.

The estimator based on the work by Xing and Ganju gives an unbiased estimate for the covariance but not for the correlation, especially when only a small number of blocks is available. It also has a large standard error which can be so large that a 95% confidence interval spans the entire range of possible values for the correlation. While increasing the number of blocks leads to a better performance, that is smaller bias and smaller standard error, it also means that the block length is shorter. However, a small block length is undesirable as, for example, Miller et al. (2009) have pointed out. Furthermore, van der Meulen (2005) shows that is possible to get some “eyesight” about the treatment effect with reasonable precision especially when a small block length is used.

The estimator based on the work of Zucker et al. shows a similar performance to the naïve estimator under the null hypothesis, that is the estimator is unbiased and has a similar standard error. However, under the alternative hypothesis, although the bias is smaller than the bias for the naïve estimator, it can still be noticeably biased. This is especially the case if the assumptions about the differences between the true group means are incorrect. However, if the sample sizes within the groups or the number of groups is not too small, the estimator leads to only slightly biased results with a standard error comparable to the one of the unblinded pooled estimator.

Overall, no estimator dominates the others uniformly. Under the null hypothesis, or under alternatives reasonably close to the null hypothesis, use of the naïve estimator is clearly recommended as it requires no additional information and its performance is nearly the same as the unblinded estimator. Under the alternative, the use of the estimator based on the work of Xing and Ganju performs best if sample sizes per group are small, only very few groups are available, and the block length used for the block randomisation is short. Otherwise the estimator based on the work of Zucker et al. can be considered if reasonably accurate estimates for the group means exist.

## Conflict of interest

*The authors have declared no conflict of interest*.

## Supporting information

As a service to our authors and readers, this journal provides supporting information supplied by the authors. Such materials are peer reviewed and may be re‐organized for online delivery, but are not copy‐edited or typeset. Technical support issues arising from supporting information (other than missing files) should be addressed to the authors.

**Figure 1**. Mean (±s.e.) for the estimate of the correlation coefficient.
**Figure 2**. Mean (±s.e.) for the estimate of the correlation coefficient for Example 1.
**Figure 3**. Mean (±s.e.) for the estimate of the correlation coefficient for Example 2.

Supporting Information

## Appendix 1

### 1.1

**Table A.1:** Simulation results for different scenarios under *H*_0_ (Example 1)

		$n_{g}=6$			$n_{g}=24$		
		G=2	G=3	G=5	G=2	G=3	G=5
	$r_{sr}$	−0.79 (0.13)	−0.79 (0.10)	−0.79 (0.07)	−0.80 (0.05)	−0.80 (0.04)	−0.80 (0.03)
	$r_{pool}$	−0.78 (0.14)	−0.79 (0.10)	−0.79 (0.08)	−0.80 (0.05)	−0.80 (0.04)	−0.80 (0.03)
ρ=−0.8	*r*_naïve_	−0.79 (0.13)	−0.79 (0.10)	−0.80 (0.07)	−0.80 (0.05)	−0.80 (0.04)	−0.80 (0.03)
	$r_{XG,B=2}$	−0.60 (0.80)	−0.58 (0.81)	−0.58 (0.82)	−0.60 (0.80)	−0.58 (0.81)	−0.59 (0.81)
	$r_{XG,B=n_{g}}$	−0.76 (0.23)	−0.76 (0.23)	−0.77 (0.22)	−0.79 (0.08)	−0.79 (0.08)	−0.79 (0.08)
	$r_{Z1,\delta =0}$	−0.79 (0.12)	−0.79 (0.09)	−0.80 (0.07)	−0.80 (0.05)	−0.80 (0.04)	−0.80 (0.03)
	$r_{Z2,\delta =0}$	−0.79 (0.13)	−0.79 (0.10)	−0.80 (0.07)	−0.80 (0.05)	−0.80 (0.04)	−0.80 (0.03)
	$r_{Z1,\delta \neq 0}$	−1.03 (0.20)	−1.02 (0.18)	−1.00 (0.07)	−0.99 (0.05)	−0.99 (0.04)	−0.99 (0.03)
	$r_{Z2,\delta \neq 0}$	−0.79 (0.13)	−0.79 (0.10)	−0.80 (0.07)	−0.80 (0.05)	−0.80 (0.04)	−0.80 (0.03)
	$r_{sr}$	0.00 (0.30)	−0.00 (0.24)	0.00 (0.19)	0.00 (0.15)	0.00 (0.12)	0.00 (0.09)
	$r_{pool}$	−0.01 (0.32)	0.01 (0.26)	−0.00 (0.20)	−0.00 (0.15)	0.00 (0.12)	−0.00 (0.09)
ρ=0	*r*_naïve_	−0.01 (0.30)	0.01 (0.24)	−0.00 (0.19)	−0.00 (0.15)	0.00 (0.12)	−0.00 (0.09)
	$r_{XG,B=2}$	0.01 (1.00)	0.00 (1.00)	0.00 (1.00)	0.01 (1.00)	0.01 (1.00)	−0.02 (1.00)
	$r_{XG,B=n_{g}}$	−0.00 (0.45)	0.01 (0.45)	0.00 (0.45)	−0.00 (0.21)	−0.00 (0.21)	−0.00 (0.21)
	$r_{Z1,\delta =0}$	−0.01 (0.29)	0.01 (0.24)	−0.00 (0.18)	−0.00 (0.15)	−0.00 (0.12)	−0.00 (0.09)
	$r_{Z2,\delta =0}$	−0.01 (0.30)	0.01 (0.24)	−0.00 (0.19)	−0.00 (0.15)	−0.00 (0.12)	−0.00 (0.09)
	$r_{Z1,\delta \neq 0}$	−0.08 (0.36)	−0.06 (0.29)	−0.07 (0.22)	−0.06 (0.17)	−0.06 (0.14)	−0.06 (0.11)
	$r_{Z2,\delta \neq 0}$	−0.01 (0.30)	0.01 (0.24)	−0.00 (0.19)	−0.00 (0.15)	−0.00 (0.12)	−0.00 (0.09)
	$r_{sr}$	0.78 (0.13)	0.79 (0.10)	0.79 (0.07)	0.80 (0.05)	0.80 (0.04)	0.80 (0.03)
	$r_{pool}$	0.78 (0.13)	0.79 (0.10)	0.79 (0.08)	0.80 (0.06)	0.80 (0.04)	0.80 (0.03)
ρ=+0.8	*r*_naïve_	0.78 (0.13)	0.79 (0.10)	0.79 (0.07)	0.80 (0.05)	0.80 (0.04)	0.80 (0.03)
	$r_{XG,B=2}$	0.59 (0.81)	0.60 (0.80)	0.58 (0.82)	0.58 (0.81)	0.59 (0.81)	0.59 (0.81)
	$r_{XG,B=n_{g}}$	0.77 (0.22)	0.77 (0.22)	0.76 (0.23)	0.79 (0.08)	0.79 (0.08)	0.79 (0.08)
	$r_{Z1,\delta =0}$	0.79 (0.12)	0.79 (0.09)	0.79 (0.07)	0.80 (0.05)	0.80 (0.04)	0.80 (0.03)
	$r_{Z2,\delta =0}$	0.78 (0.13)	0.79 (0.10)	0.79 (0.07)	0.80 (0.05)	0.80 (0.04)	0.80 (0.03)
	$r_{Z1,\delta \neq 0}$	0.88 (0.23)	0.88 (0.11)	0.87 (0.07)	0.87 (0.06)	0.87 (0.05)	0.87 (0.03)
	$r_{Z2,\delta \neq 0}$	0.78 (0.13)	0.79 (0.10)	0.79 (0.07)	0.80 (0.05)	0.80 (0.04)	0.80 (0.03)

Notes. Number in brackets give standard errors

**Table A.2:** Simulation results for different scenarios under *H*_1_ (Example 2)

		$n_{g}=6$			$n_{g}=24$		
		G=2	G=3	G=5	G=2	G=3	G=5
	$r_{sr}$	−0.42 (0.23)	−0.53 (0.17)	−0.59 (0.12)	−0.43 (0.10)	−0.54 (0.08)	−0.60 (0.06)
	$r_{pool}$	−0.79 (0.14)	−0.79 (0.10)	−0.79 (0.08)	−0.80 (0.06)	−0.80 (0.04)	−0.80 (0.03)
ρ=−0.8	*r*_naïve_	−0.39 (0.23)	−0.51 (0.17)	−0.59 (0.11)	−0.43 (0.10)	−0.54 (0.08)	−0.60 (0.05)
	$r_{XG,B=2}$	−0.59 (0.81)	−0.59 (0.81)	−0.59 (0.81)	−0.60 (0.80)	−0.59 (0.81)	−0.59 (0.81)
	$r_{XG,B=n_{g}}$	−0.77 (0.23)	−0.77 (0.22)	−0.76 (0.23)	−0.79 (0.08)	−0.79 (0.08)	−0.79 (0.08)
	$r_{Z1,\delta =0}$	−0.98 (1.72)	−0.87 (0.33)	−0.83 (0.17)	−0.82 (0.14)	−0.81 (0.10)	−0.81 (0.07)
	$r_{Z2,\delta =0}$	−0.87 (0.47)	−0.82 (0.17)	−0.81 (0.11)	−0.81 (0.10)	−0.80 (0.07)	−0.80 (0.05)
	$r_{Z1,\delta \neq 0}$	−0.56 (0.53)	−0.59 (0.30)	−0.61 (0.19)	−0.53 (0.13)	−0.57 (0.10)	−0.60 (0.07)
	$r_{Z2,\delta \neq 0}$	−0.62 (0.29)	−0.67 (0.17)	−0.70 (0.11)	−0.62 (0.10)	−0.67 (0.07)	−0.70 (0.05)
	$r_{sr}$	0.20 (0.29)	0.14 (0.24)	0.11 (0.18)	0.20 (0.14)	0.14 (0.11)	0.11 (0.09)
	$r_{pool}$	0.00 (0.32)	0.00 (0.26)	0.00 (0.20)	0.00 (0.15)	0.00 (0.12)	−0.00 (0.09)
ρ=0	*r*_naïve_	0.22 (0.28)	0.15 (0.24)	0.11 (0.18)	0.20 (0.14)	0.15 (00.11)	0.11 (0.09)
	$r_{XG,B=2}$	0.02 (1.00)	0.01 (1.00)	0.00 (1.00)	0.01 (1.00)	−0.01 (1.00)	−0.01 (1.00)
	$r_{XG,B=n_{g}}$	0.00 (0.45)	−0.01 (0.45)	0.01 (0.45)	0.00 (0.21)	0.00 (0.21)	−0.00 (0.21)
	$r_{Z1,\delta =0}$	−0.13 (1.03)	−0.07 (0.46)	−0.03 (0.26)	−0.02 (0.22)	−0.01 (0.16)	−0.01 (0.12)
	$r_{Z2,\delta =0}$	−0.07 (0.55)	−0.03 (0.30)	−0.01 (0.21)	−0.01 (0.18)	−0.00 (0.14)	−0.00 (0.10)
	$r_{Z1,\delta \neq 0}$	0.18 (0.59)	0.15 (0.32)	0.14 (0.23)	0.20 (0.18)	0.17 (0.14)	0.15 (0.11)
	$r_{Z2,\delta \neq 0}$	0.10 (0.38)	0.07 (0.27)	0.06 (0.20)	0.11 (0.16)	0.08 (0.13)	0.06 (0.10)
	$r_{sr}$	0.83 (0.10)	0.82 (0.08)	0.82 (0.06)	0.84 (0.04)	0.83 (0.04)	0.82 (0.03)
	$r_{pool}$	0.78 (0.14)	0.79 (0.11)	0.79 (0.08)	0.80 (0.06)	0.80 (0.04)	0.80 (0.03)
ρ=+0.8	*r*_naïve_	0.83 (0.10)	0.82 (0.08)	0.82 (0.06)	0.84 (0.04)	0.83 (0.04)	0.82 (0.03)
	$r_{XG,B=2}$	0.59 (0.81)	0.58 (0.82)	0.61 (0.79)	0.59 (0.82)	0.59 (0.80)	0.59 (0.81)
	$r_{XG,B=n_{g}}$	0.76 (0.23)	0.77 (0.22)	0.76 (0.23)	0.80 (0.08)	0.79 (0.08)	0.79 (0.08)
	$r_{Z1,\delta =0}$	0.71 (0.89)	0.76 (0.23)	0.79 (0.10)	0.79 (0.08)	0.79 (0.06)	0.80 (0.04)
	$r_{Z2,\delta =0}$	0.75 (0.41)	0.78 (0.12)	0.79 (0.08)	0.79 (0.07)	0.80 (0.05)	0.80 (0.04)
	$r_{Z1,\delta \neq 0}$	0.98 (0.67)	0.93 (0.13)	0.91 (0.07)	0.93 (0.05)	0.91 (0.04)	0.90 (0.03)
	$r_{Z2,\delta \neq 0}$	0.85 (0.18)	0.82 (0.10)	0.82 (0.07)	0.85 (0.05)	0.83 (0.04)	0.82 (0.03)

Notes. Numbers in brackets give standard errors.

Tables A.1 and A.2 give a detailed summary of the simulation results for Examples 1 and 2 introduced in Section 3.3 above, respectively, and correspond to results shown in Figures 2 and 3. The tables give the mean (and standard errors) for the different correlation estimates considered, including those for $r_{\text{Z1}}$ that were omitted from the figures.
