# Supplementary material for: Blinded versus unblinded estimation of a correlation coefficient to inform interim design adaptations
Source: Biom J. 2016 Nov 25;59(2):344–57. doi: 10.1002/bimj.201500233 (PMC5412911; doi:10.1002/bimj.201500233)
Supplement: Supplementary file 1 — Figure 1. Mean (±s.e.) for the estimate of the correlation coefficient. Figure 2. Mean (±s.e.) for the estimate of the correlation coefficient for Example 1. Figure 3. Mean (±s.e.) for the estimate of the correlation coefficient for Example 2. [file BIMJ-59-344-s001.pdf]

Blinded versus unblinded estimation of the correlation coefficient

Cornelia Ursula Kunz, Nigel Stallard, Nicholas Parsons, Susan Todd, and Tim Friede

May 25, 2016

1 Additional graphs for Example 1

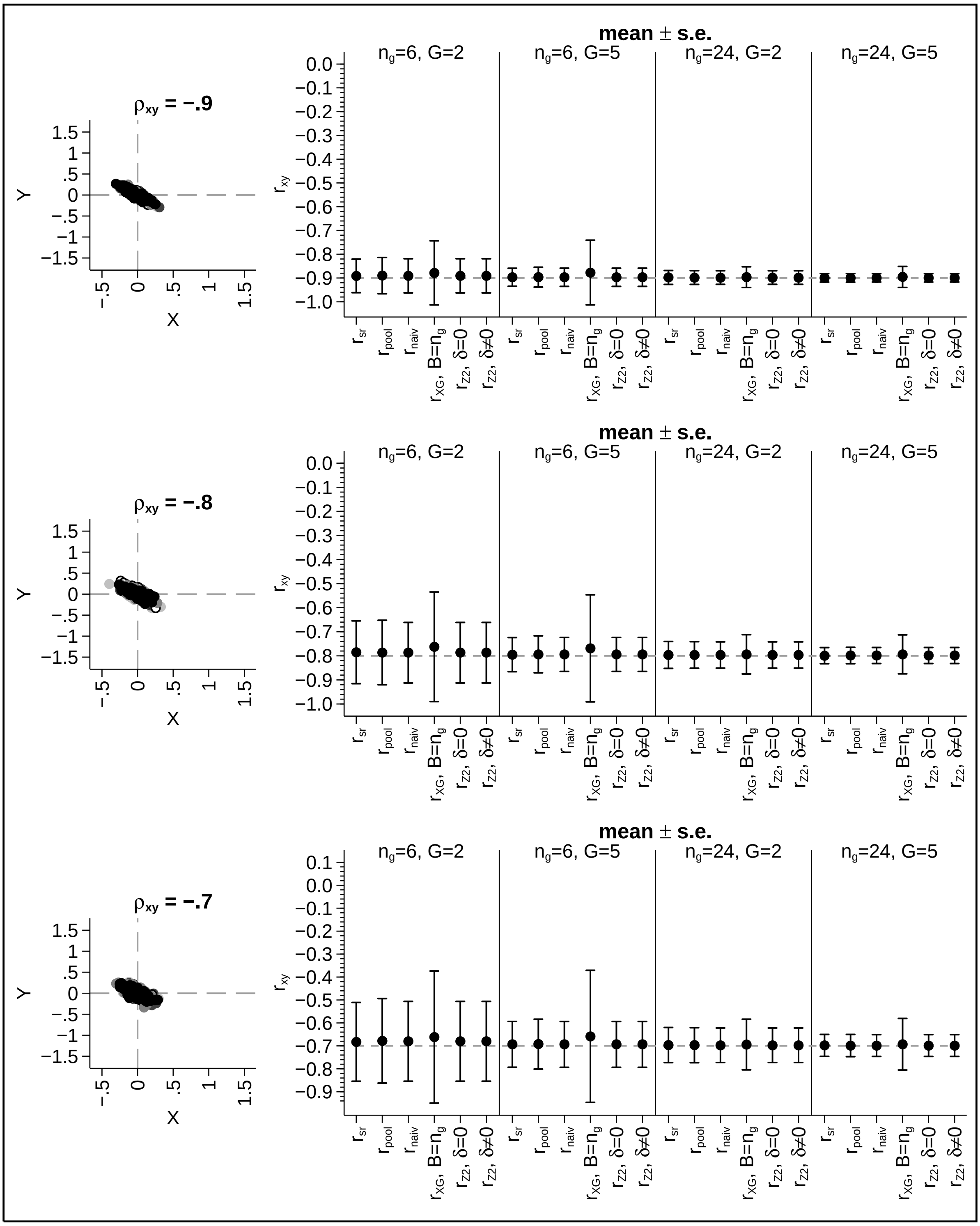

Figure 1.1: Mean (± s.e.) for the estimate of the correlation coefficient for Example 1

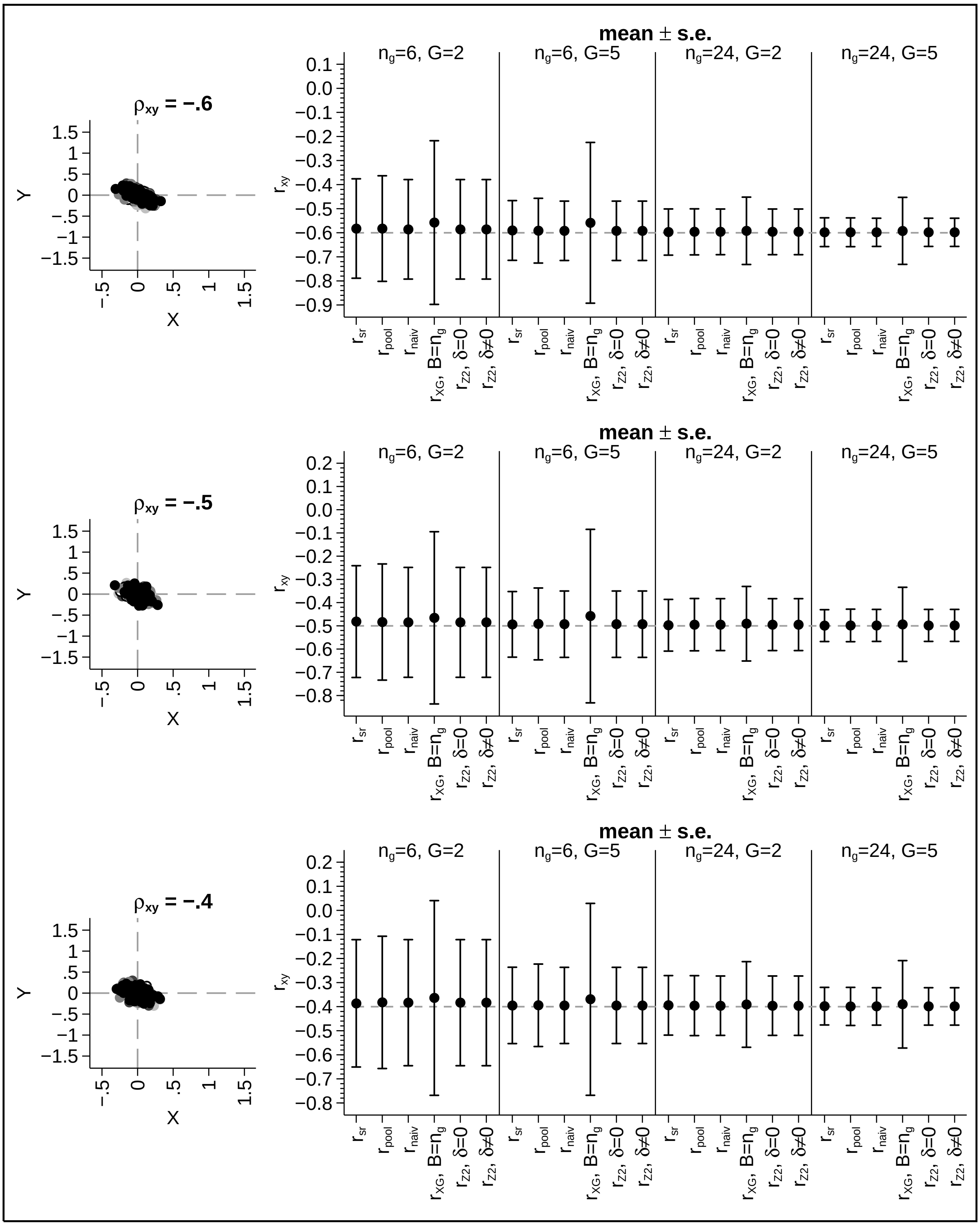

Figure 1.2: Mean (± s.e.) for the estimate of the correlation coefficient for Example 1

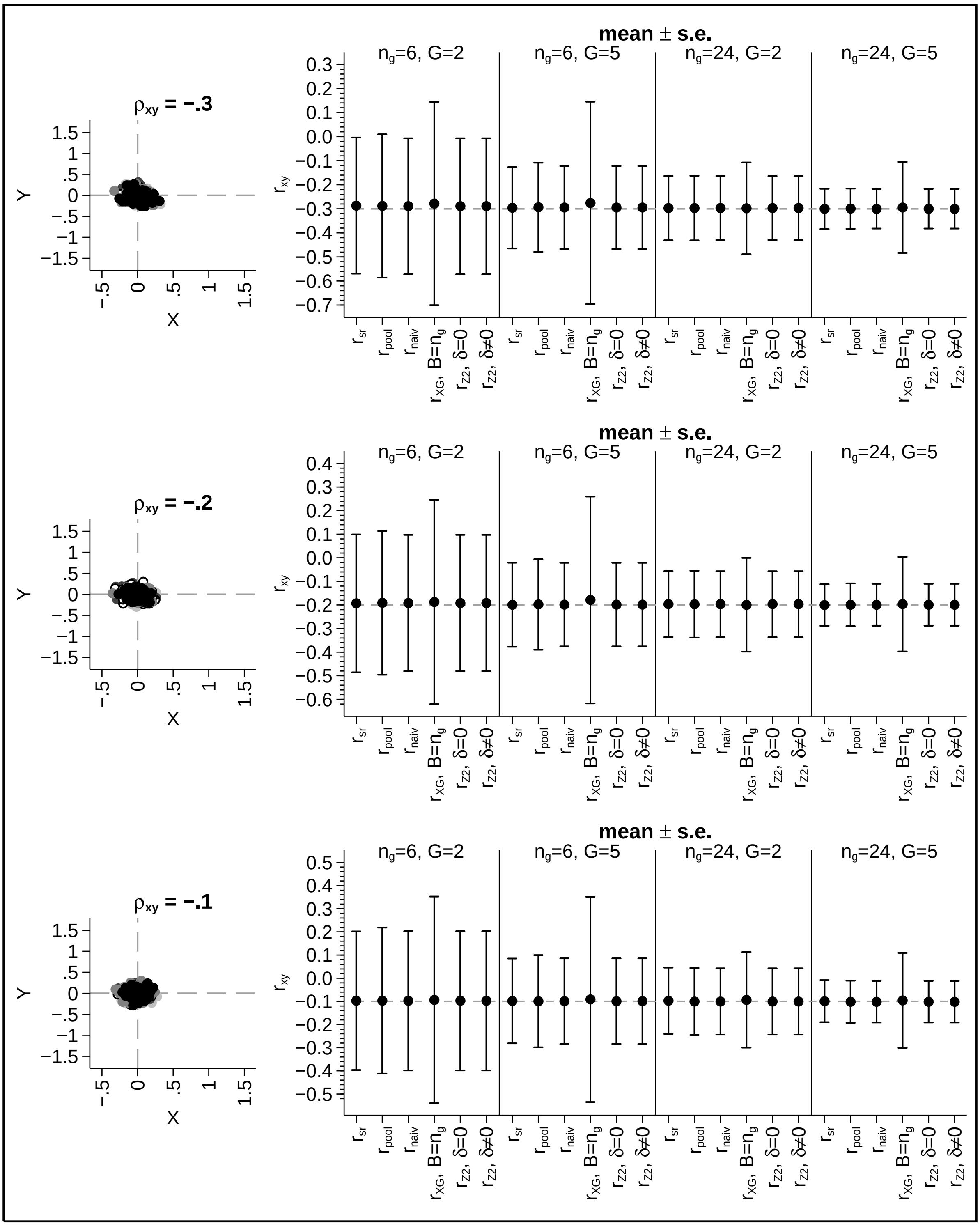

Figure 1.3: Mean (± s.e.) for the estimate of the correlation coefficient for Example 1

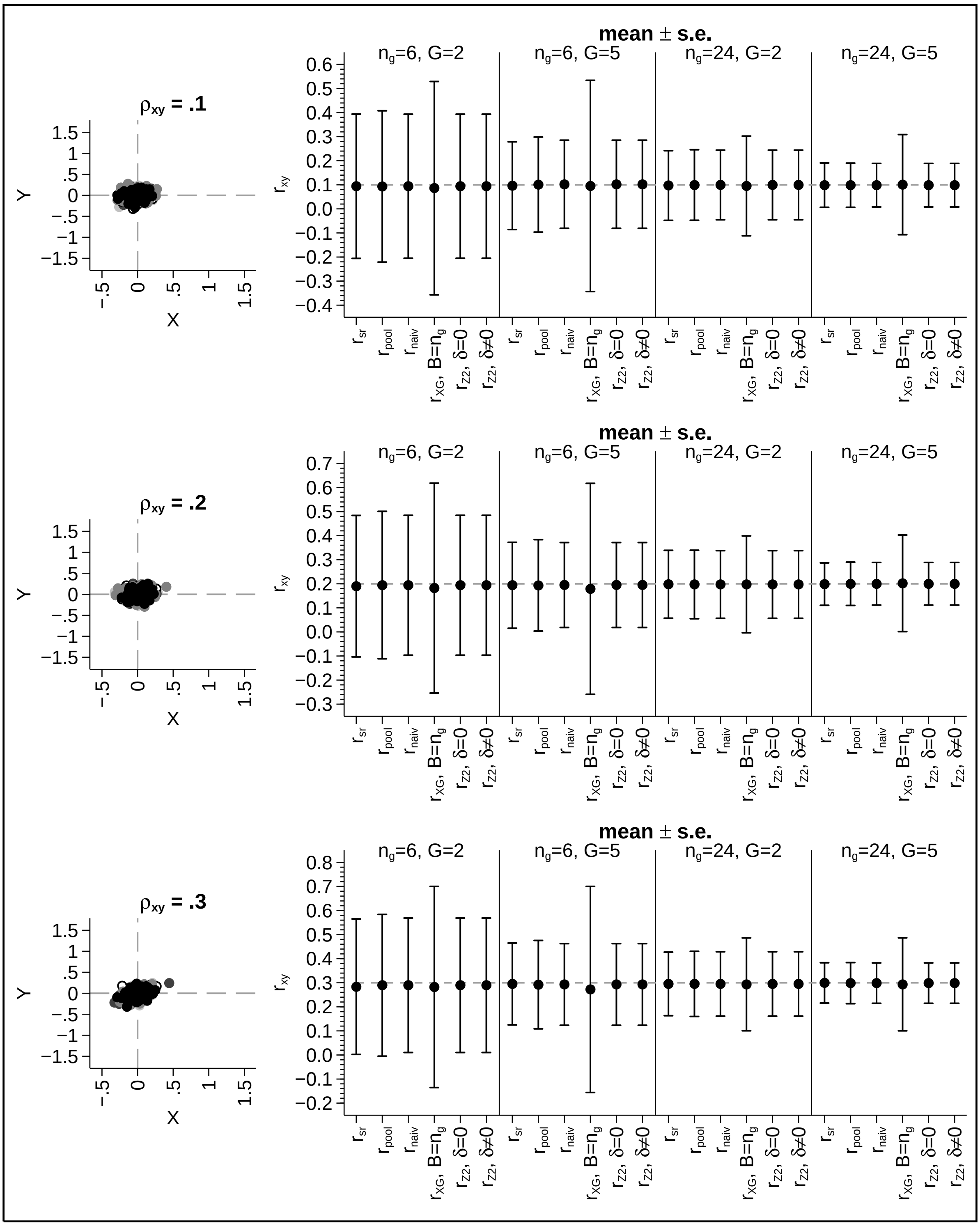

Figure 1.4: Mean (± s.e.) for the estimate of the correlation coefficient for Example 1

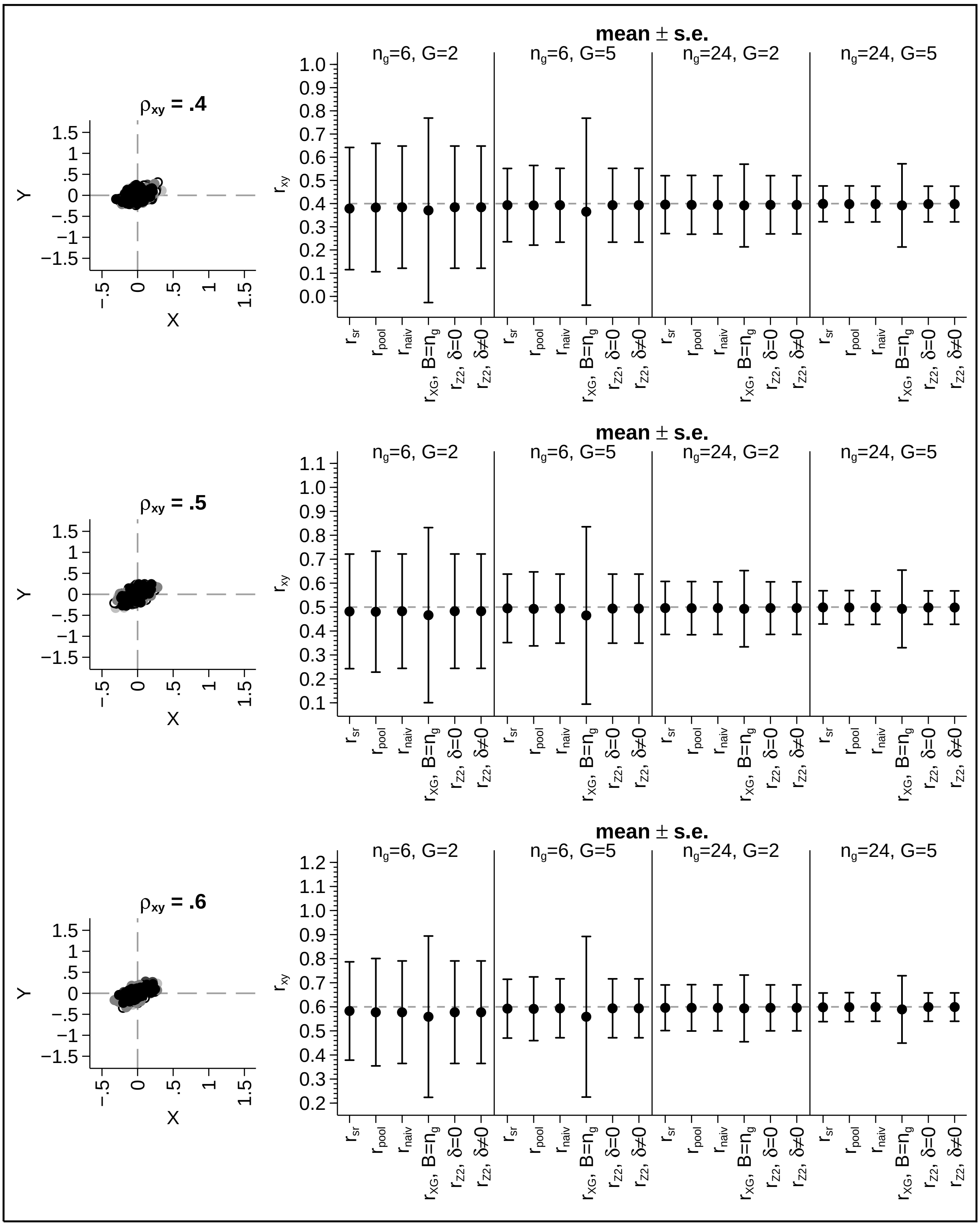

Figure 1.5: Mean (± s.e.) for the estimate of the correlation coefficient for Example 1

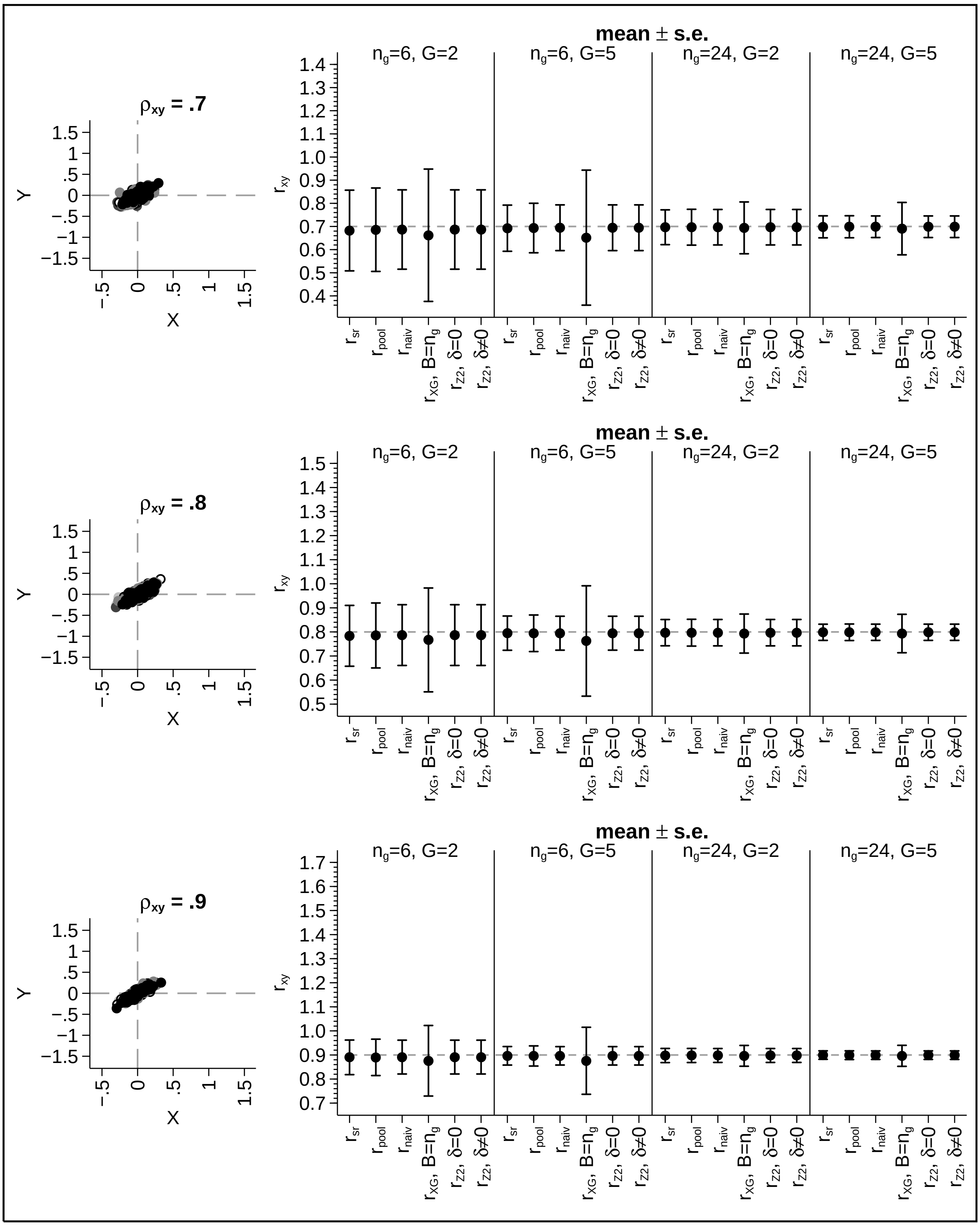

Figure 1.6: Mean (± s.e.) for the estimate of the correlation coefficient for Example 1

2 Additional graphs for Example 2

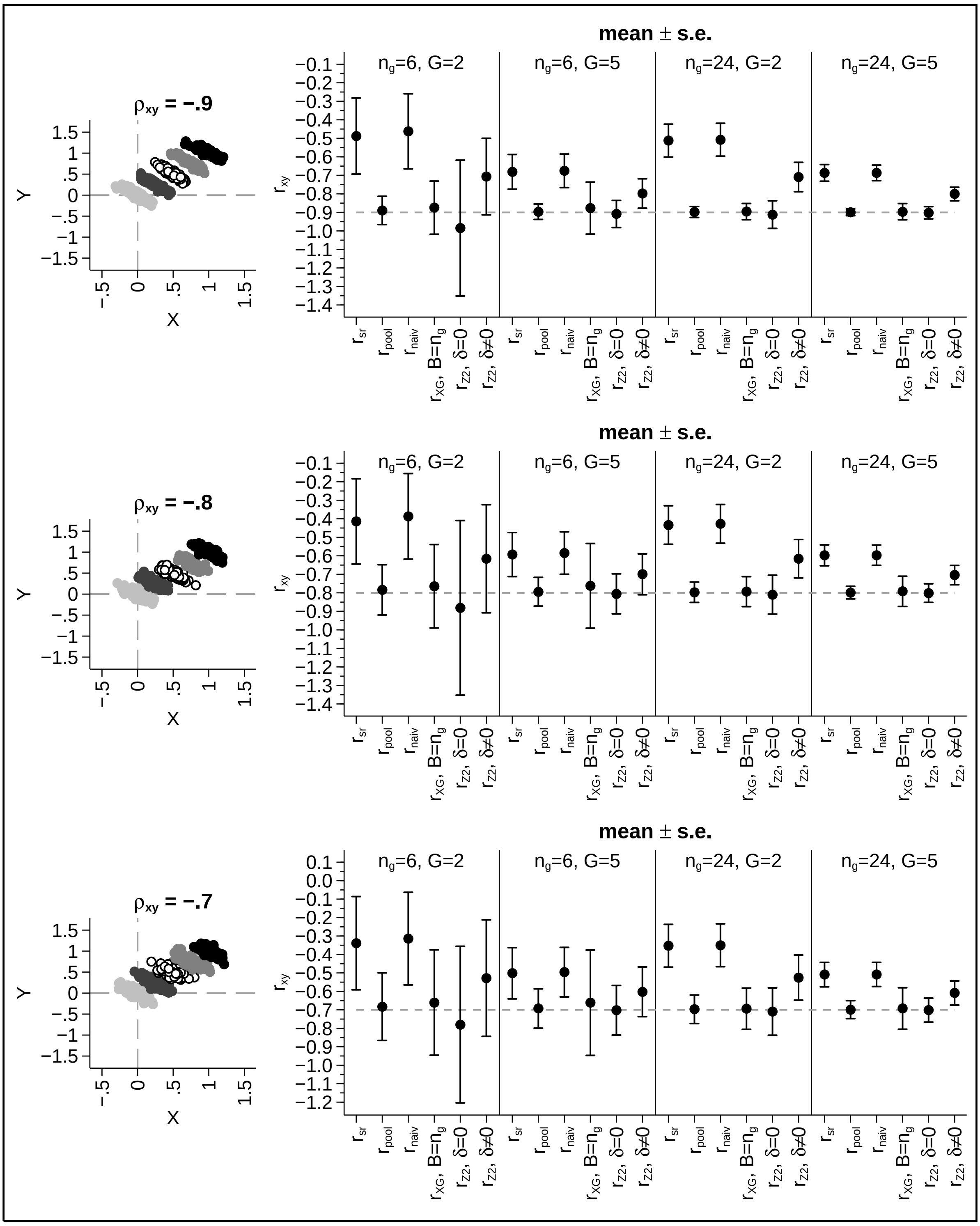

Figure 2.1: Mean (± s.e.) for the estimate of the correlation coefficient for Example 2

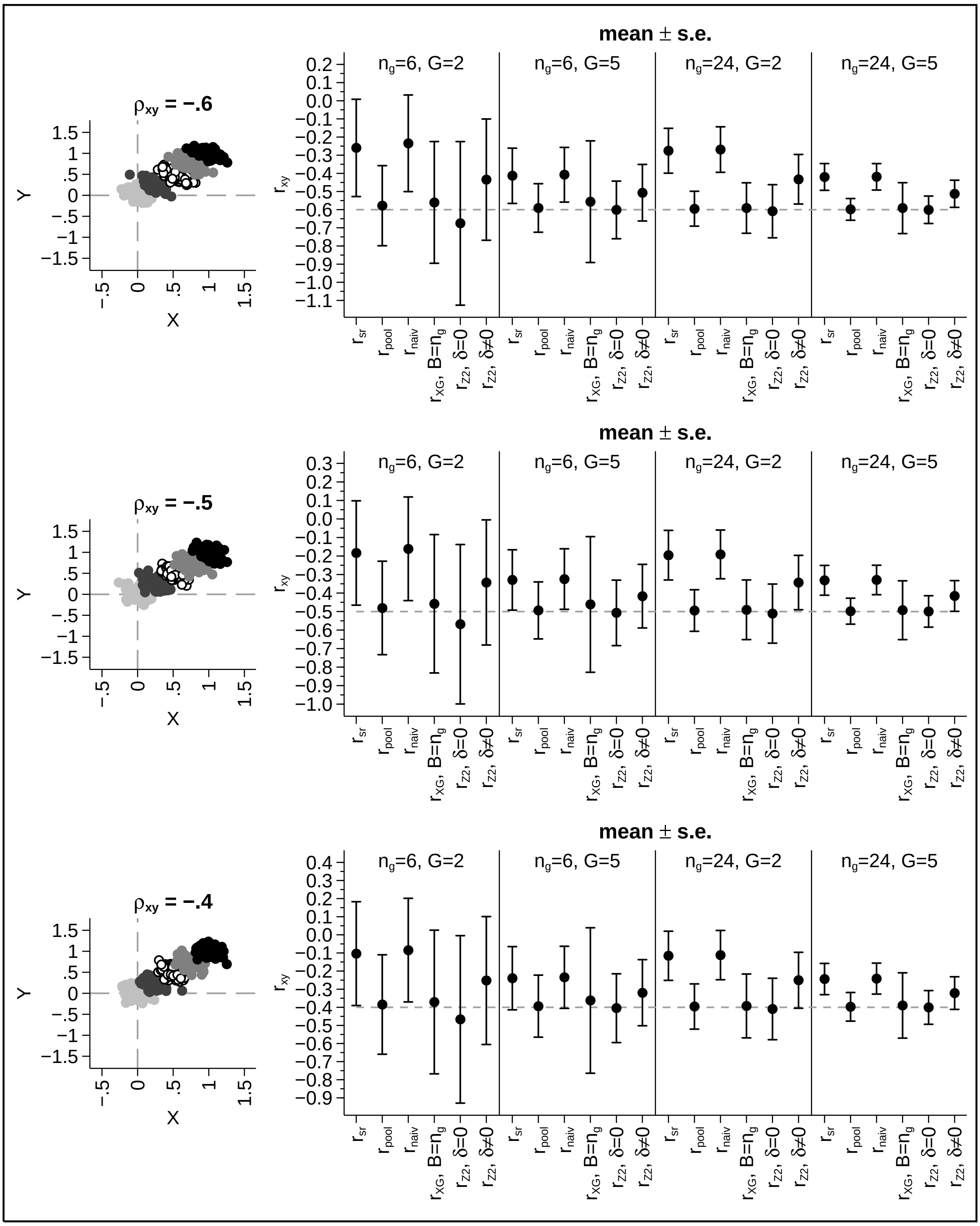

Figure 2.2: Mean (± s.e.) for the estimate of the correlation coefficient for Example 2

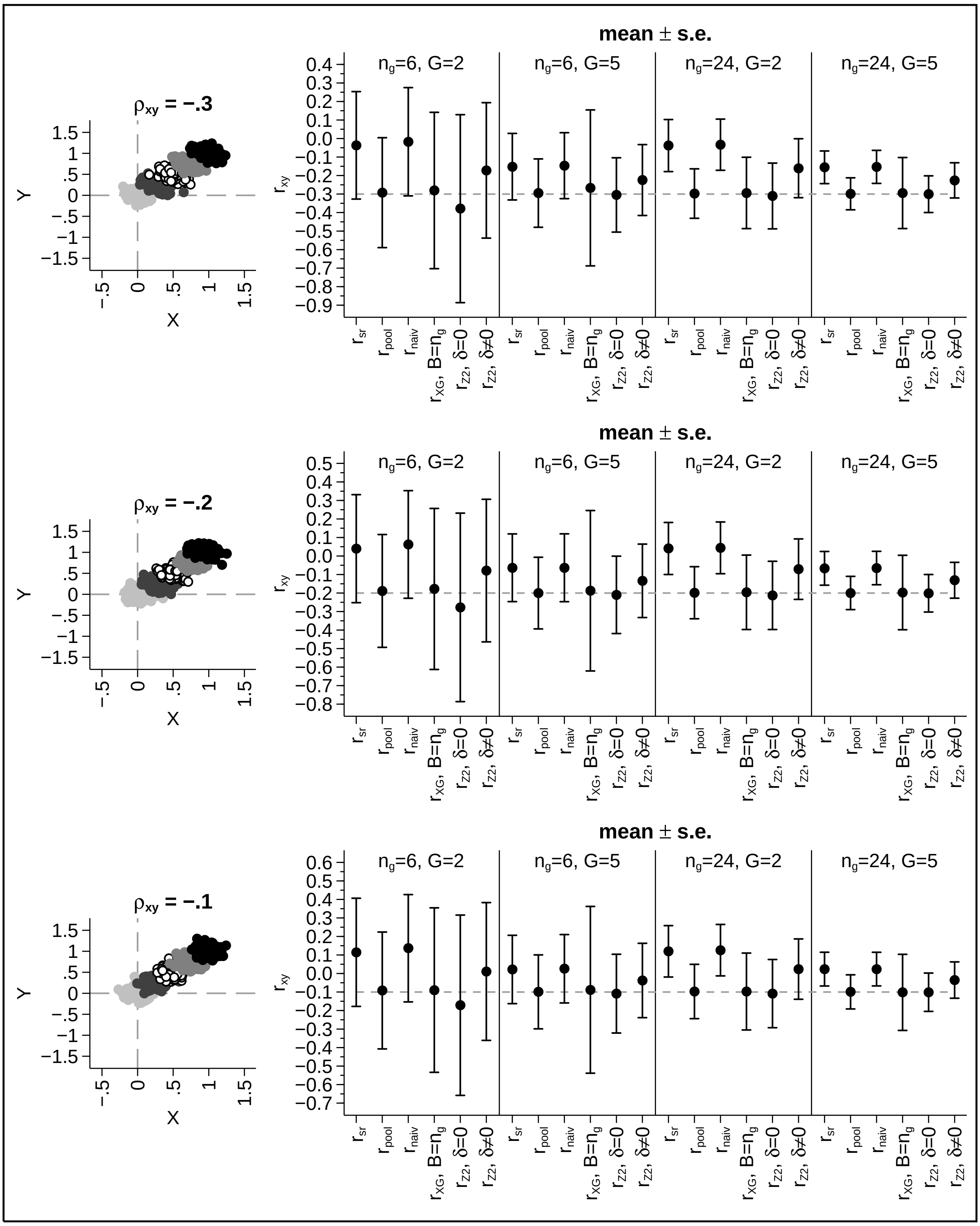

Figure 2.3: Mean (± s.e.) for the estimate of the correlation coefficient for Example 2

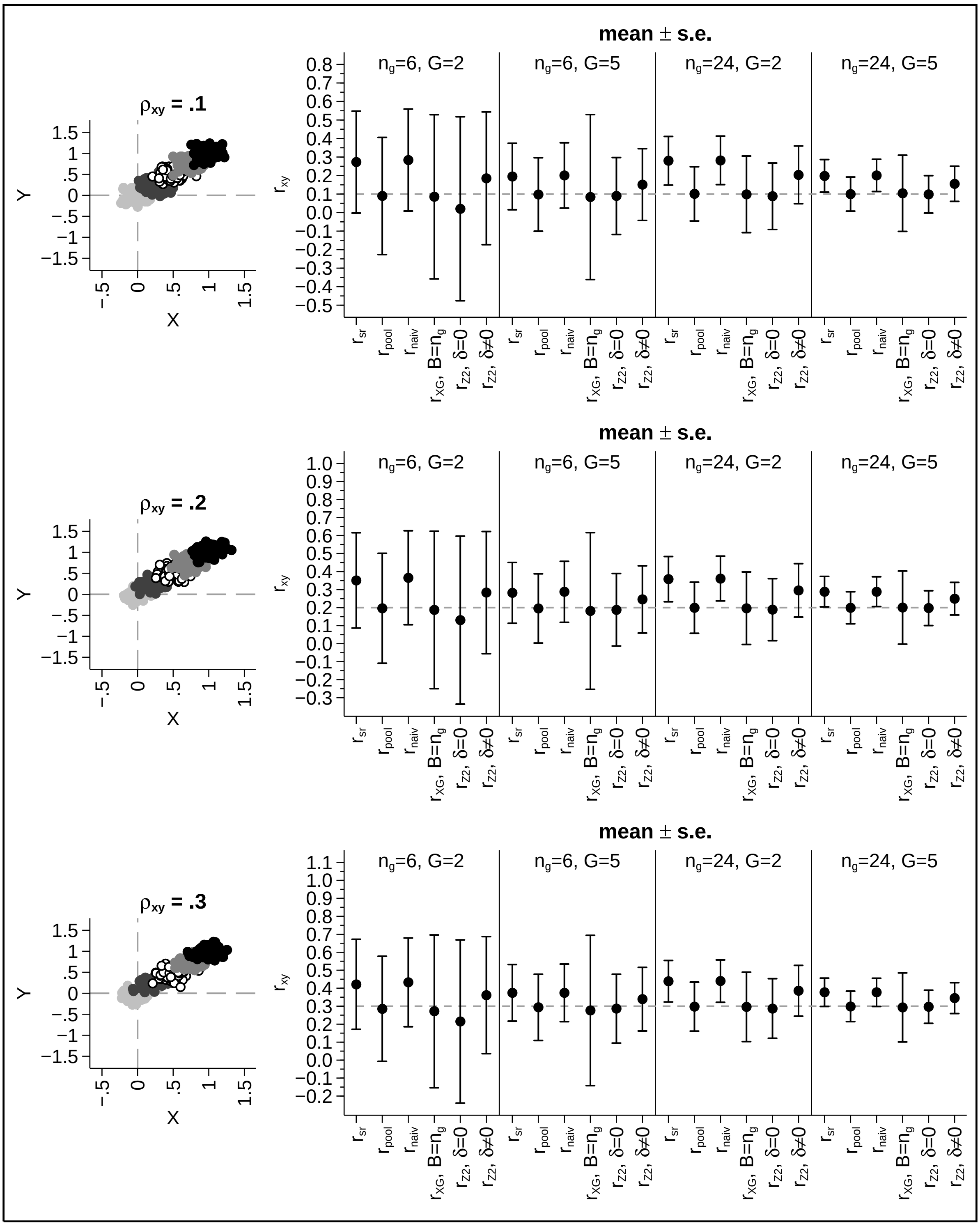

Figure 2.4: Mean (± s.e.) for the estimate of the correlation coefficient for Example 2

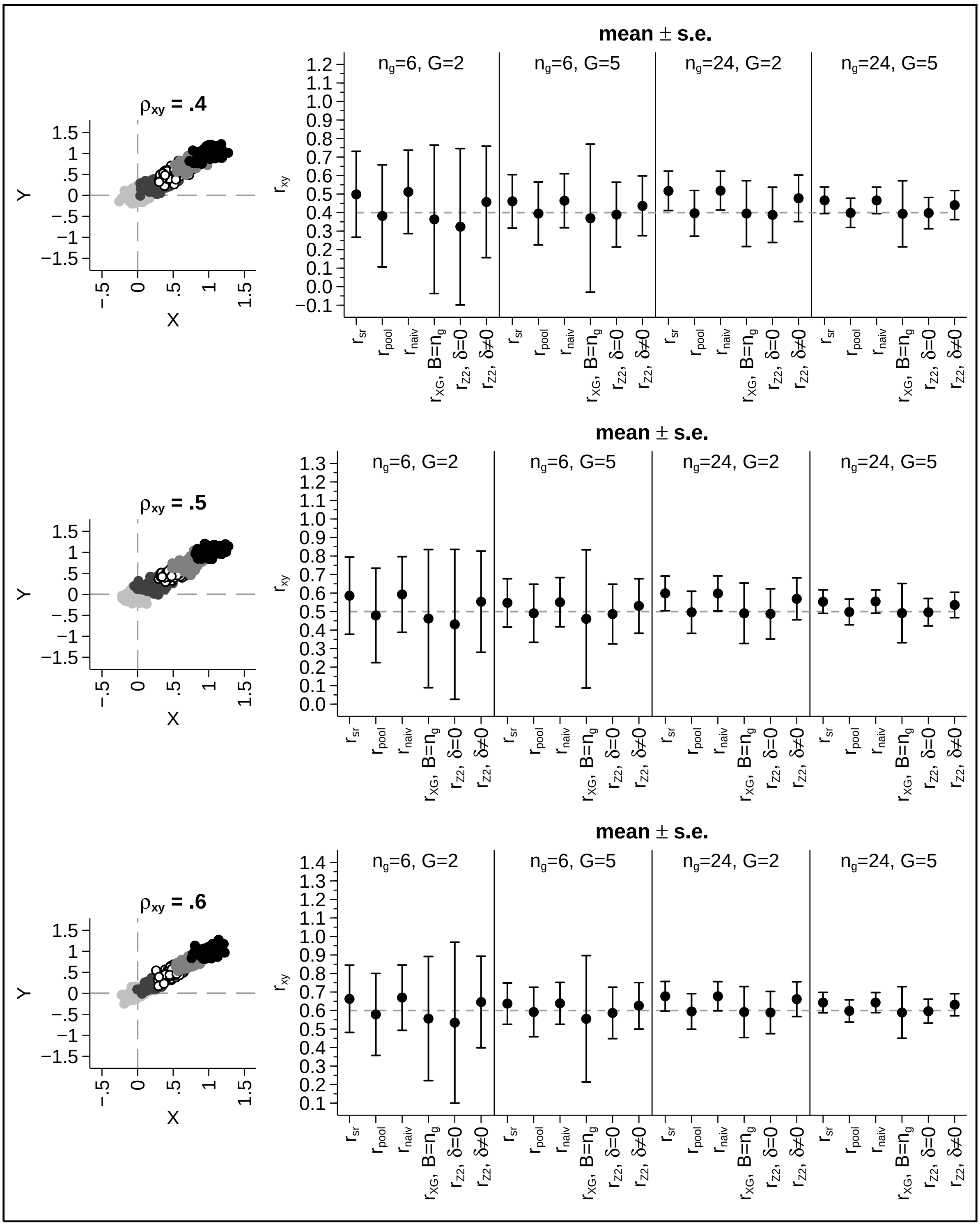

Figure 2.5: Mean (± s.e.) for the estimate of the correlation coefficient for Example 2

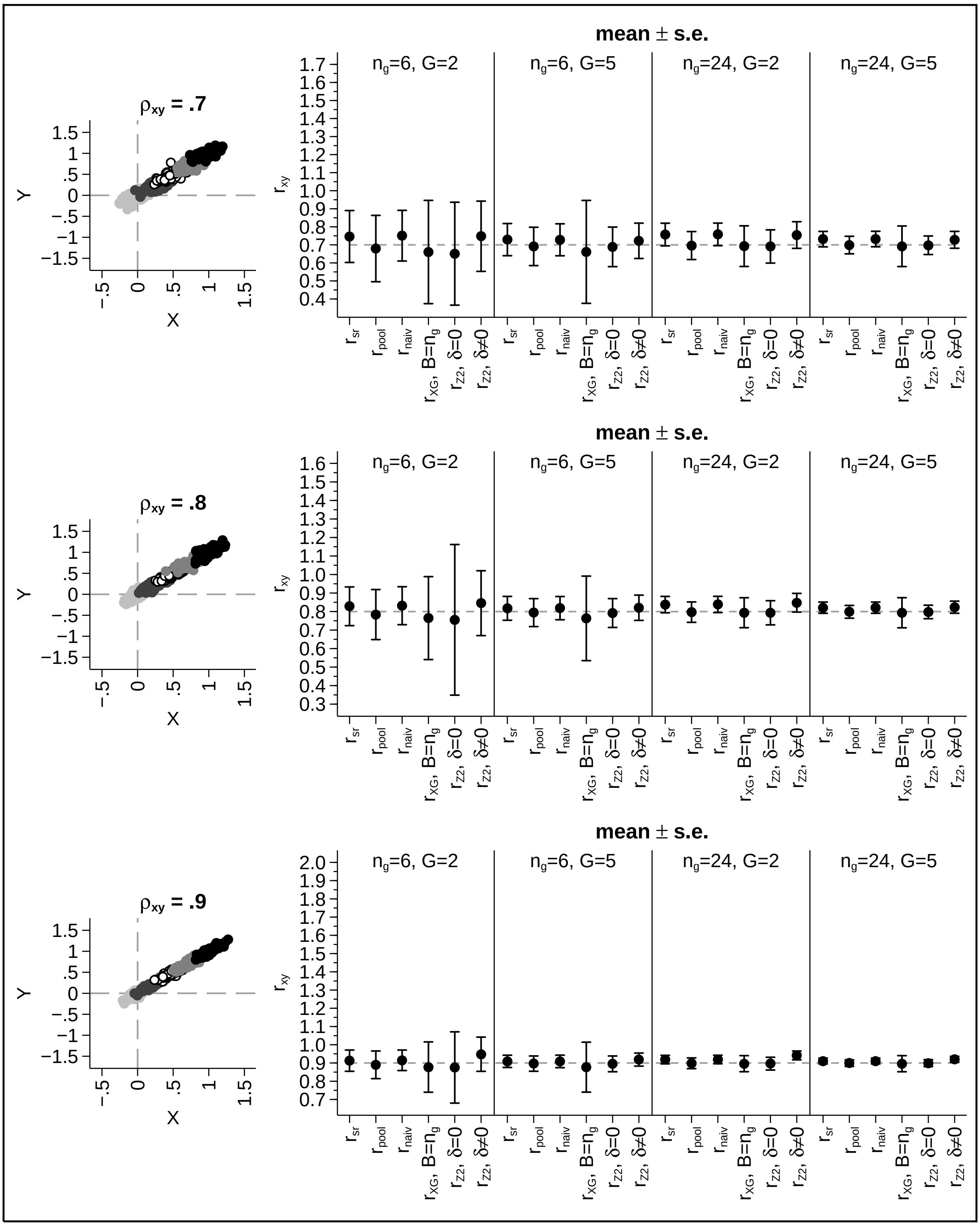

Figure 2.6: Mean (± s.e.) for the estimate of the correlation coefficient for Example 2

3 Additional graphs for different means for the two endpoints - Example 3

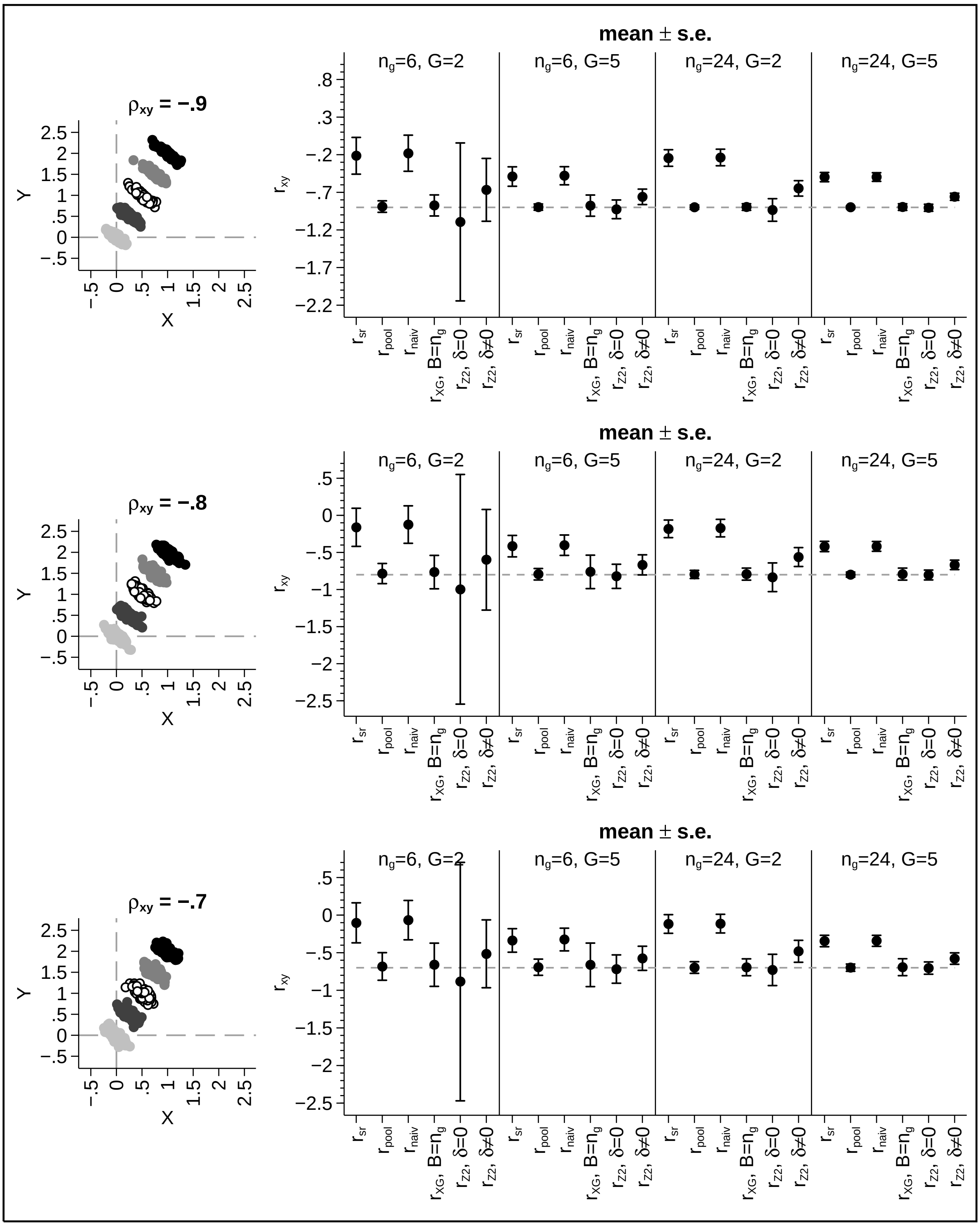

Figure 3.1: Mean (± s.e.) for the estimate of the correlation coefficient for Example 3

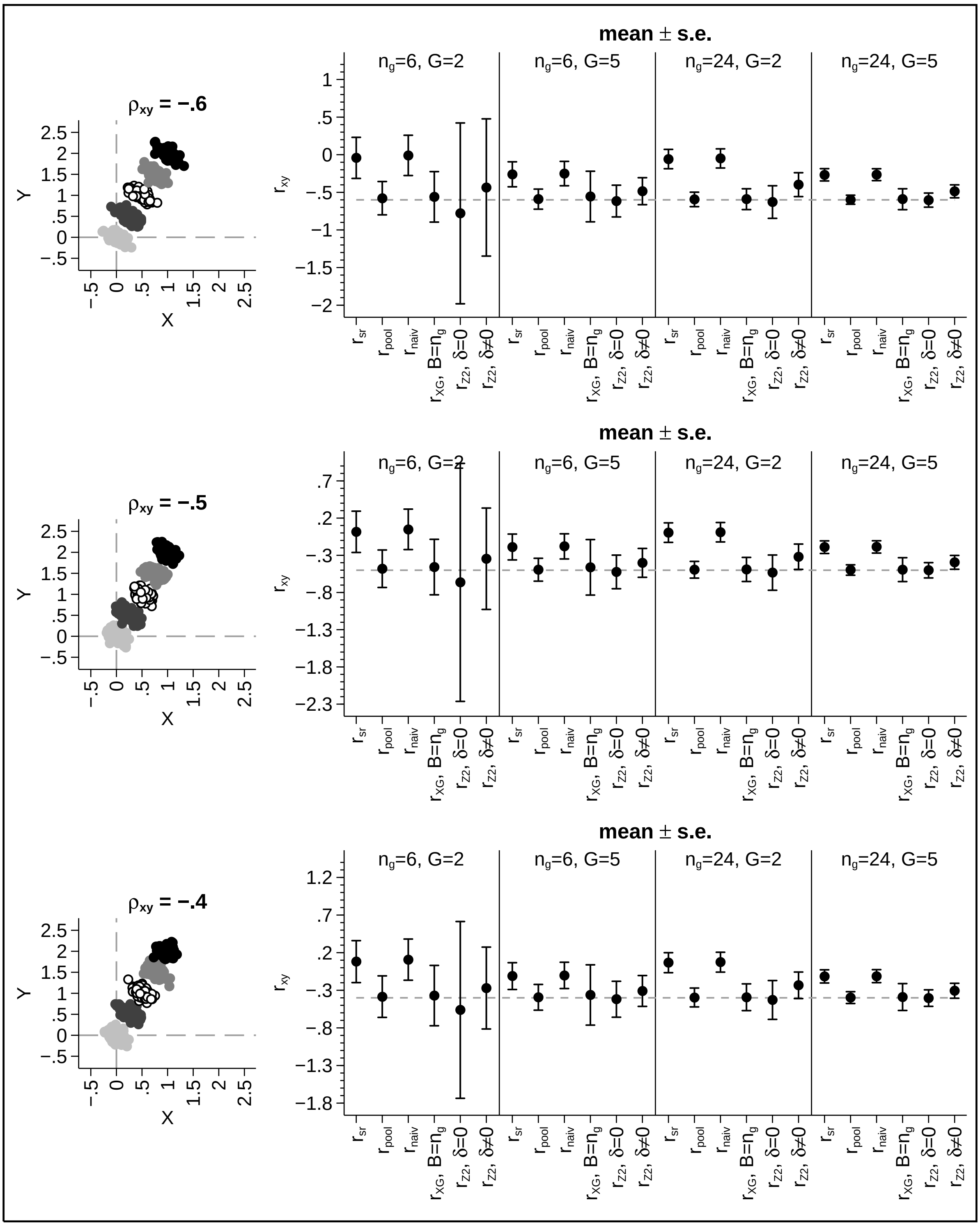

Figure 3.2: Mean (± s.e.) for the estimate of the correlation coefficient for Example 3

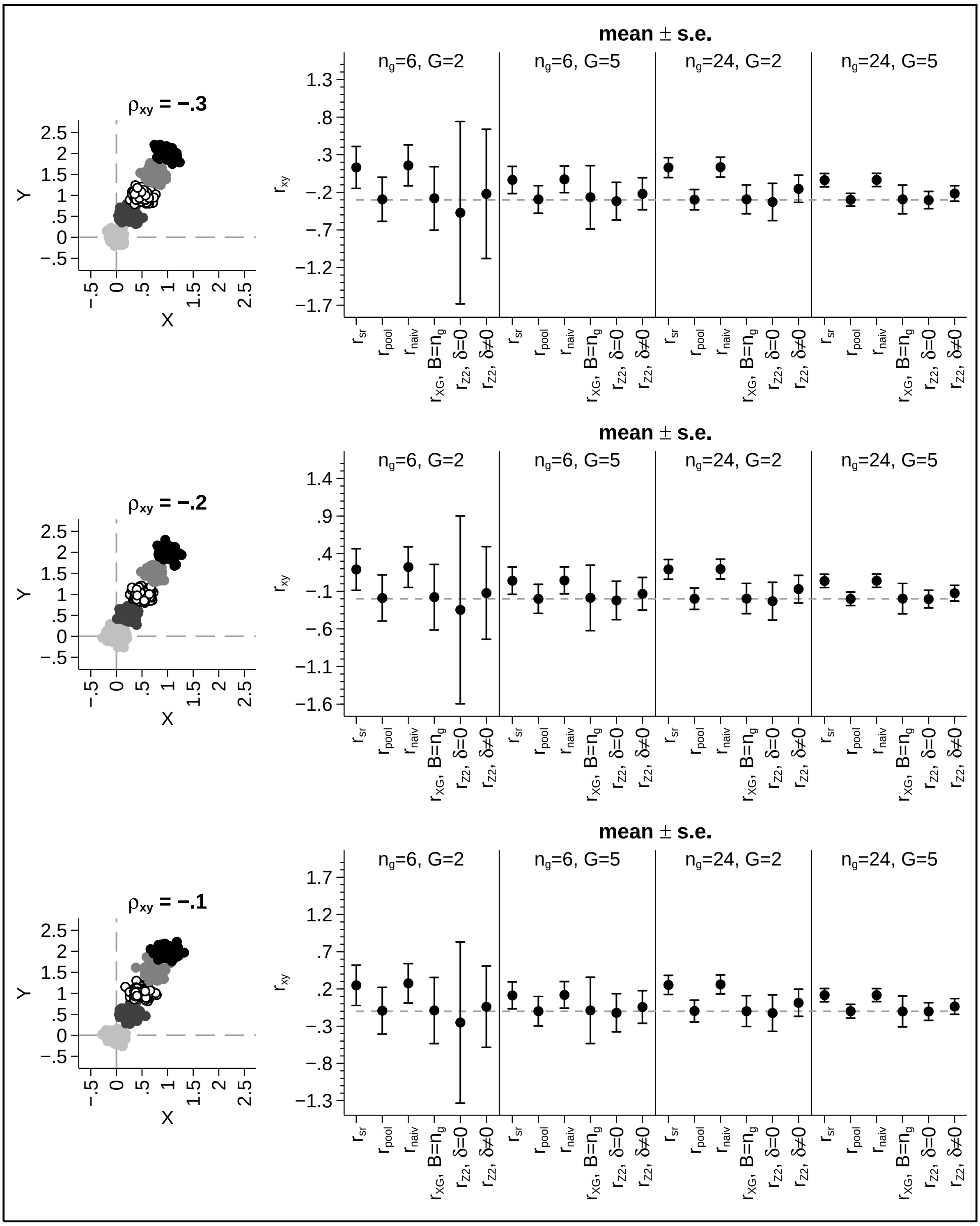

Figure 3.3: Mean (± s.e.) for the estimate of the correlation coefficient for Example 3

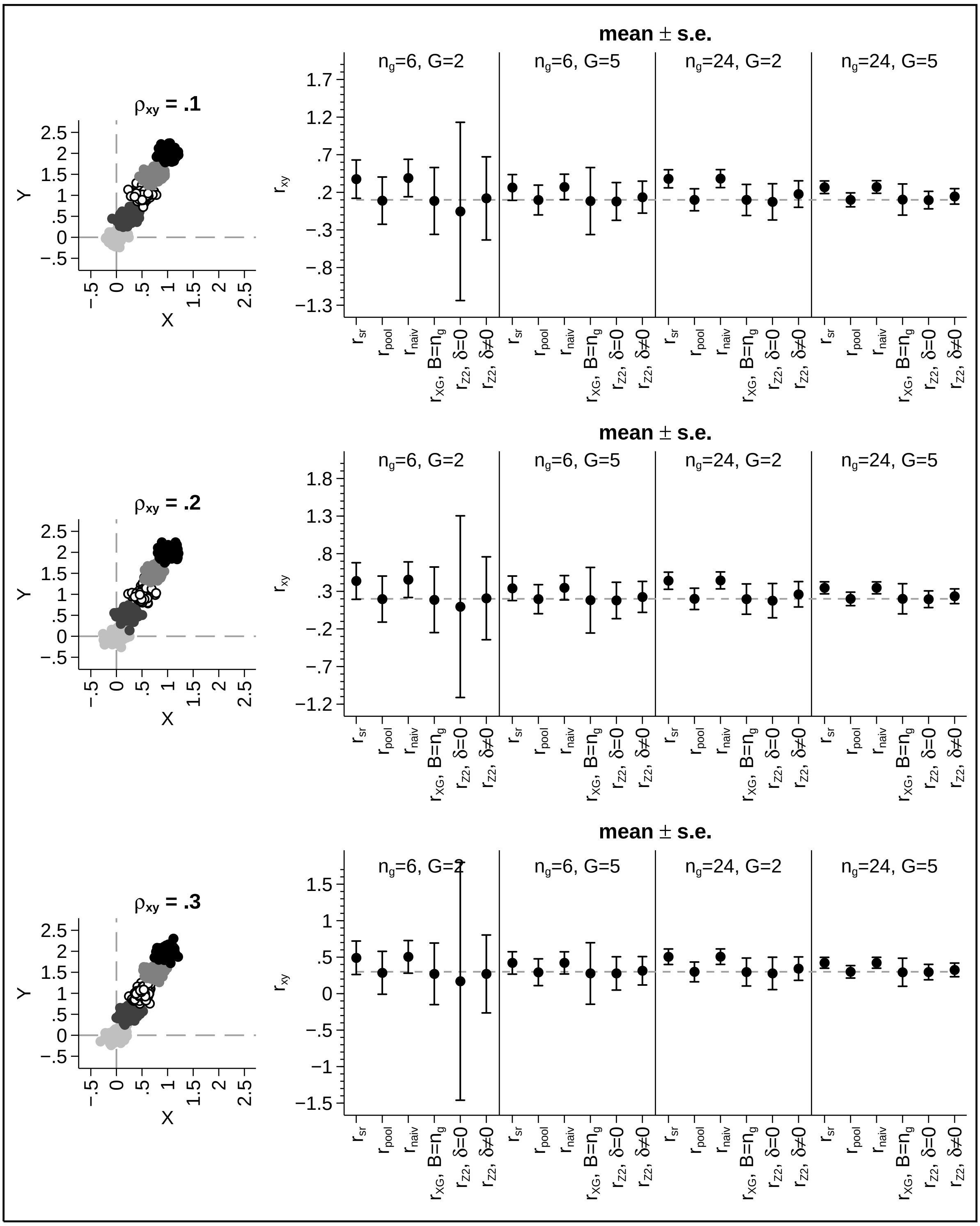

Figure 3.4: Mean ($\pm$ s.e.) for the estimate of the correlation coefficient for Example 3

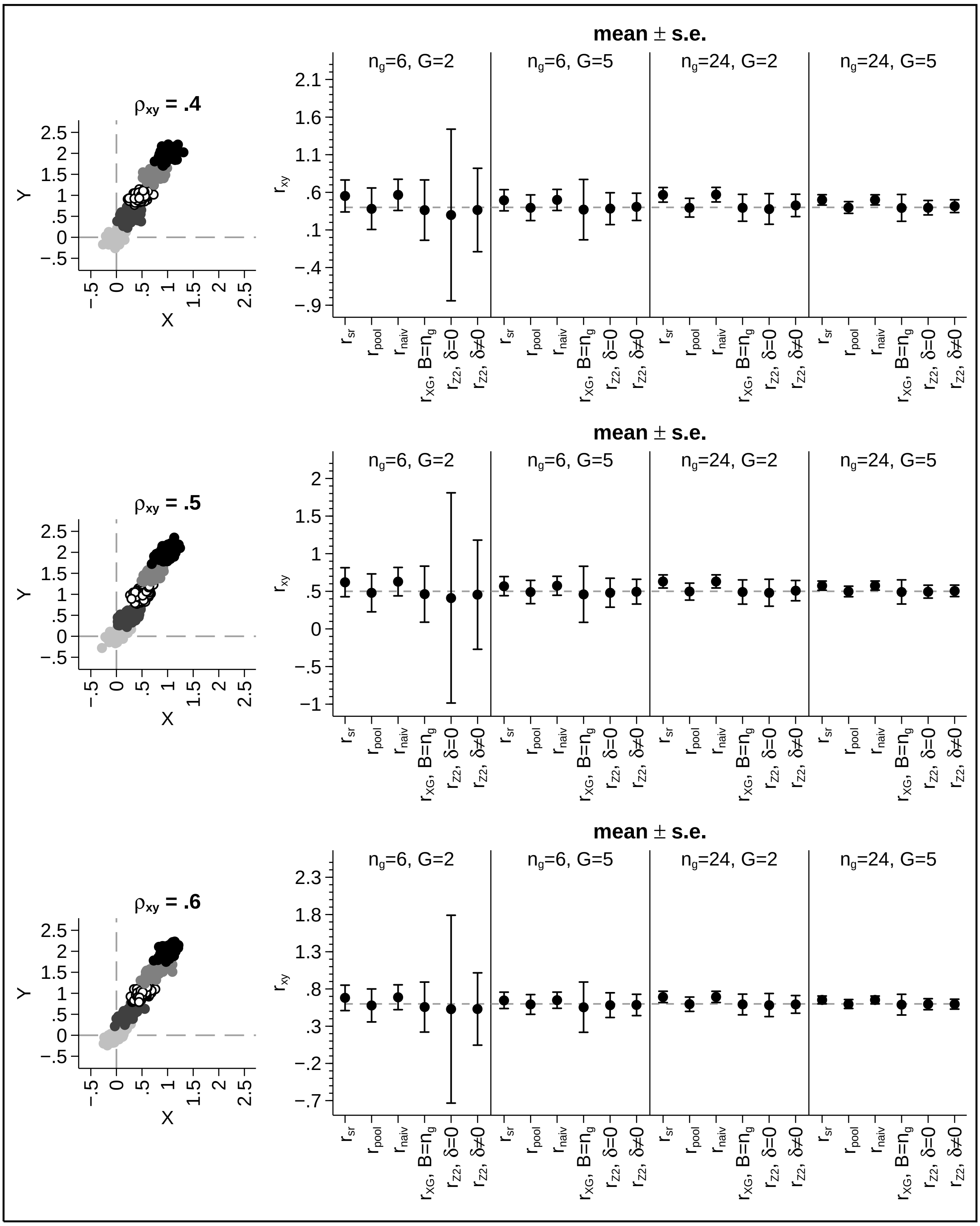

Figure 3.5: Mean (± s.e.) for the estimate of the correlation coefficient for Example 3

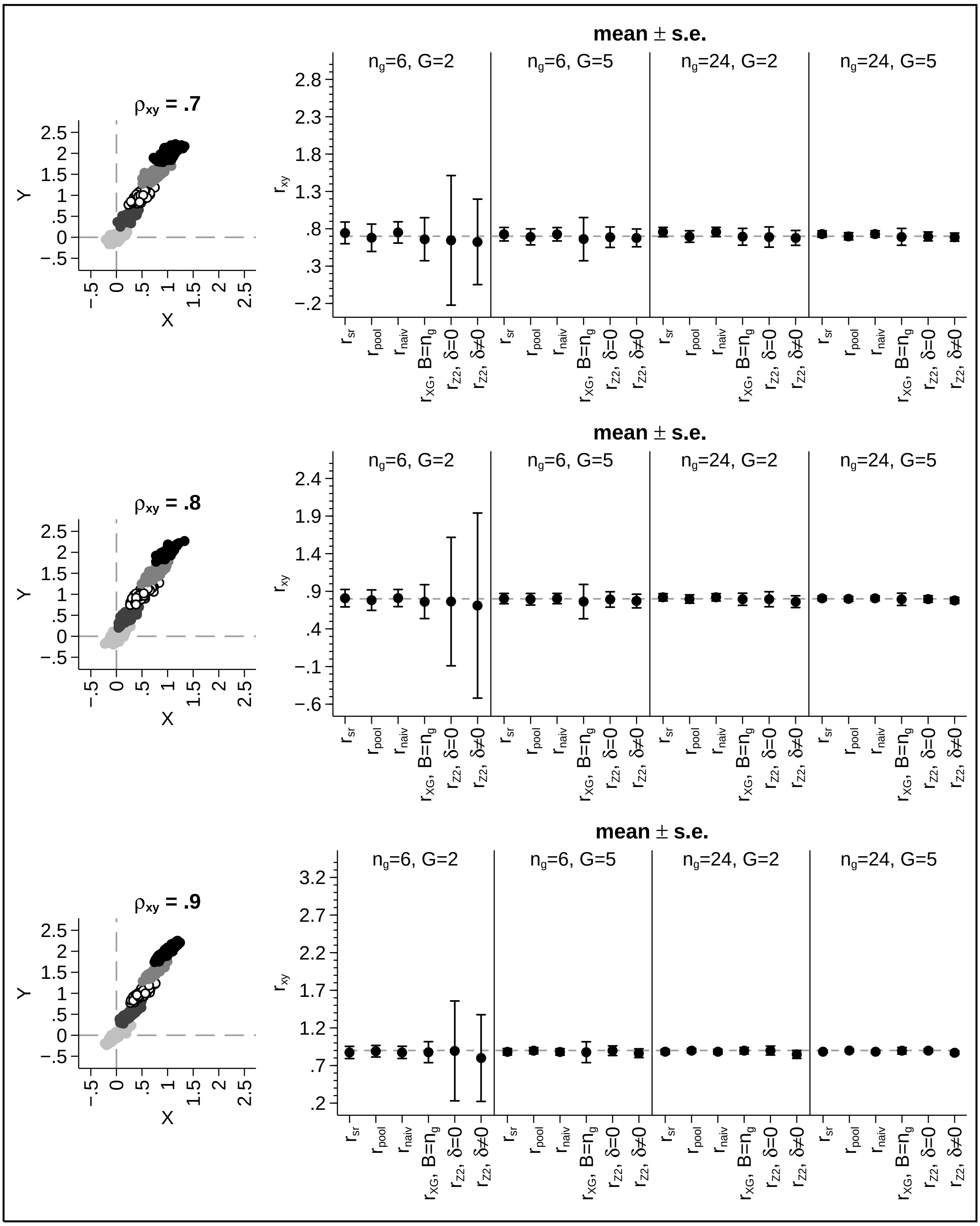

Figure 3.6: Mean (± s.e.) for the estimate of the correlation coefficient for Example 3

4 Additional graphs for different means for the two endpoints - Example 4

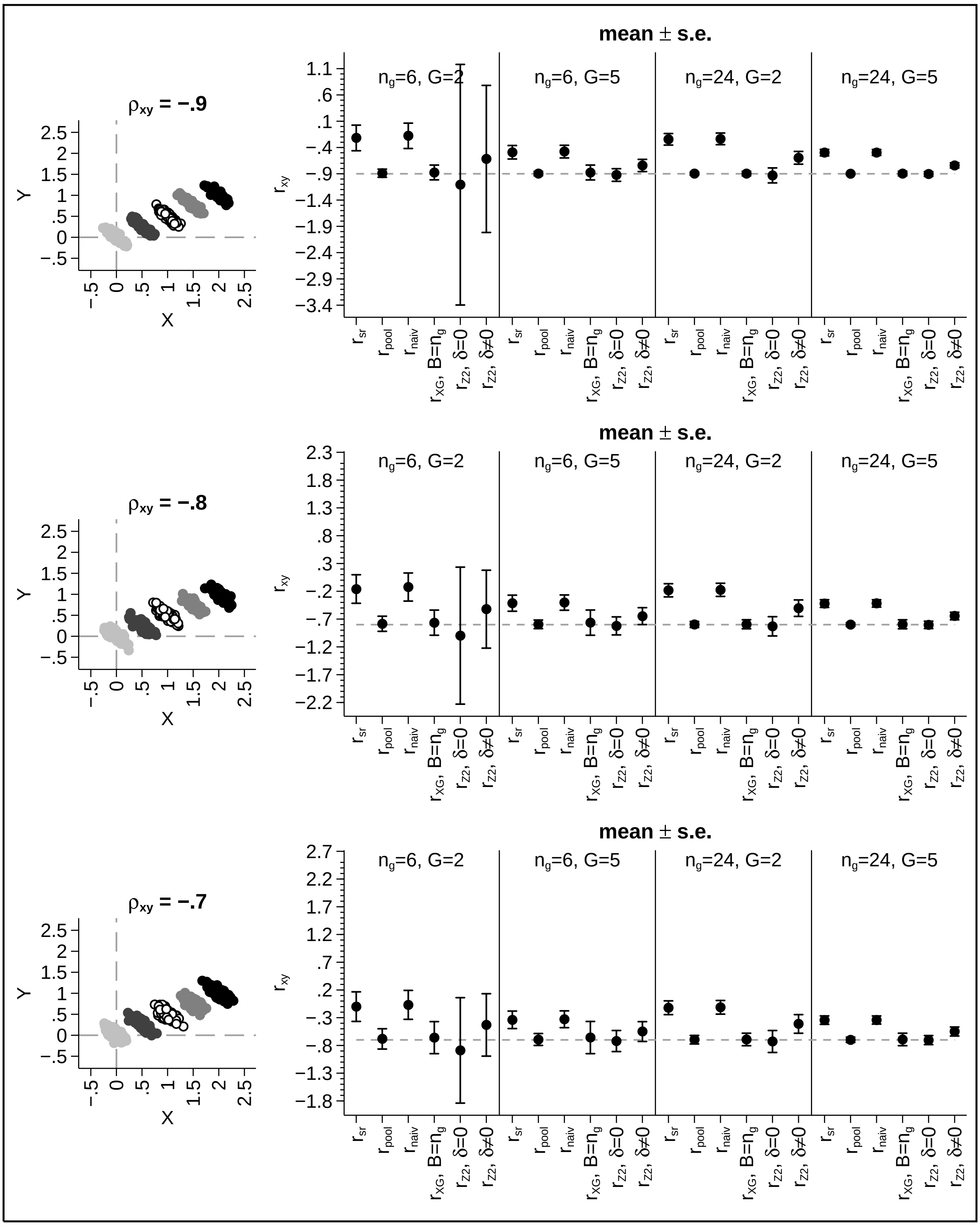

Figure 4.1: Mean (± s.e.) for the estimate of the correlation coefficient for Example 4

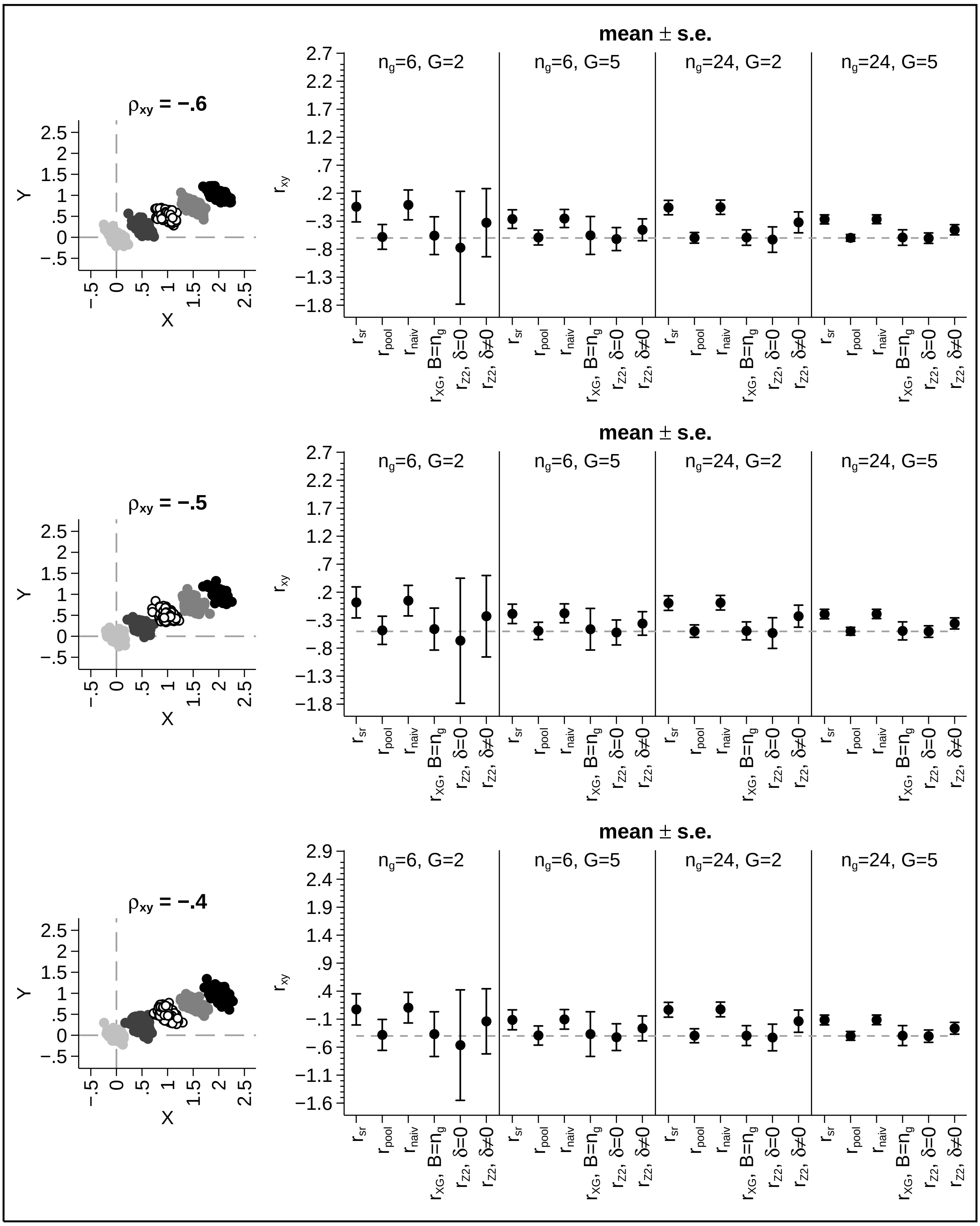

Figure 4.2: Mean (± s.e.) for the estimate of the correlation coefficient for Example 4

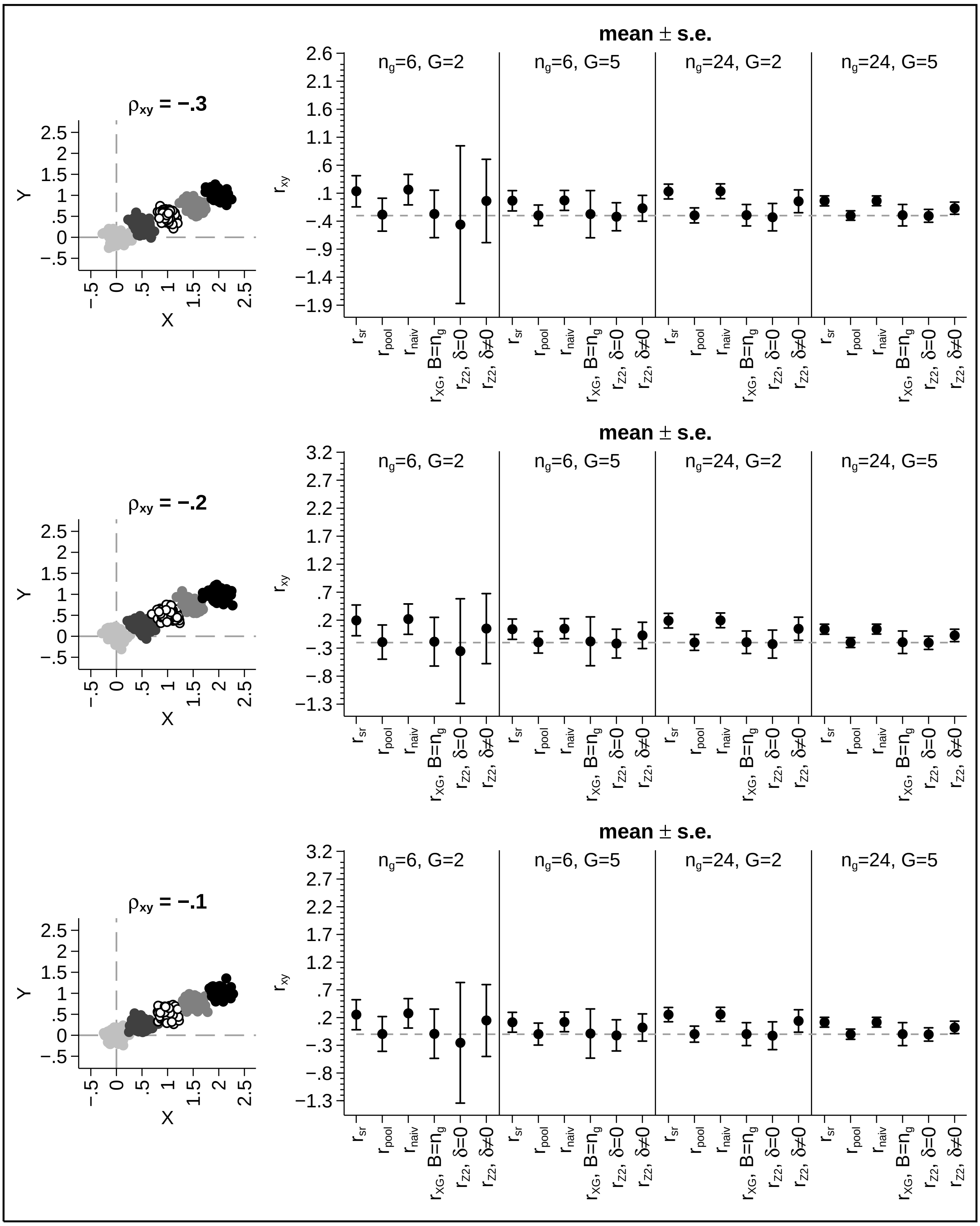

Figure 4.3: Mean (± s.e.) for the estimate of the correlation coefficient for Example 4

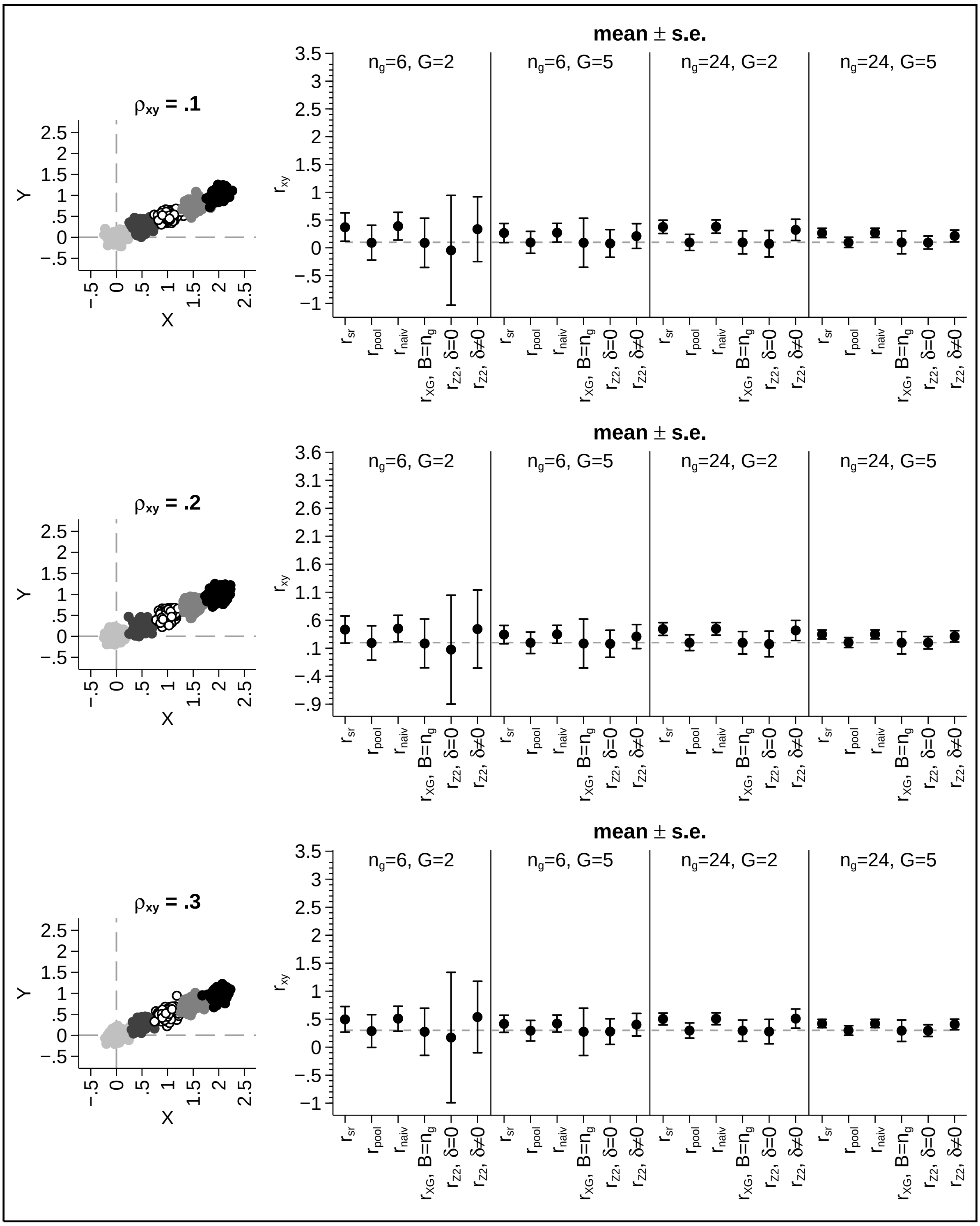

Figure 4.4: Mean (± s.e.) for the estimate of the correlation coefficient for Example 4

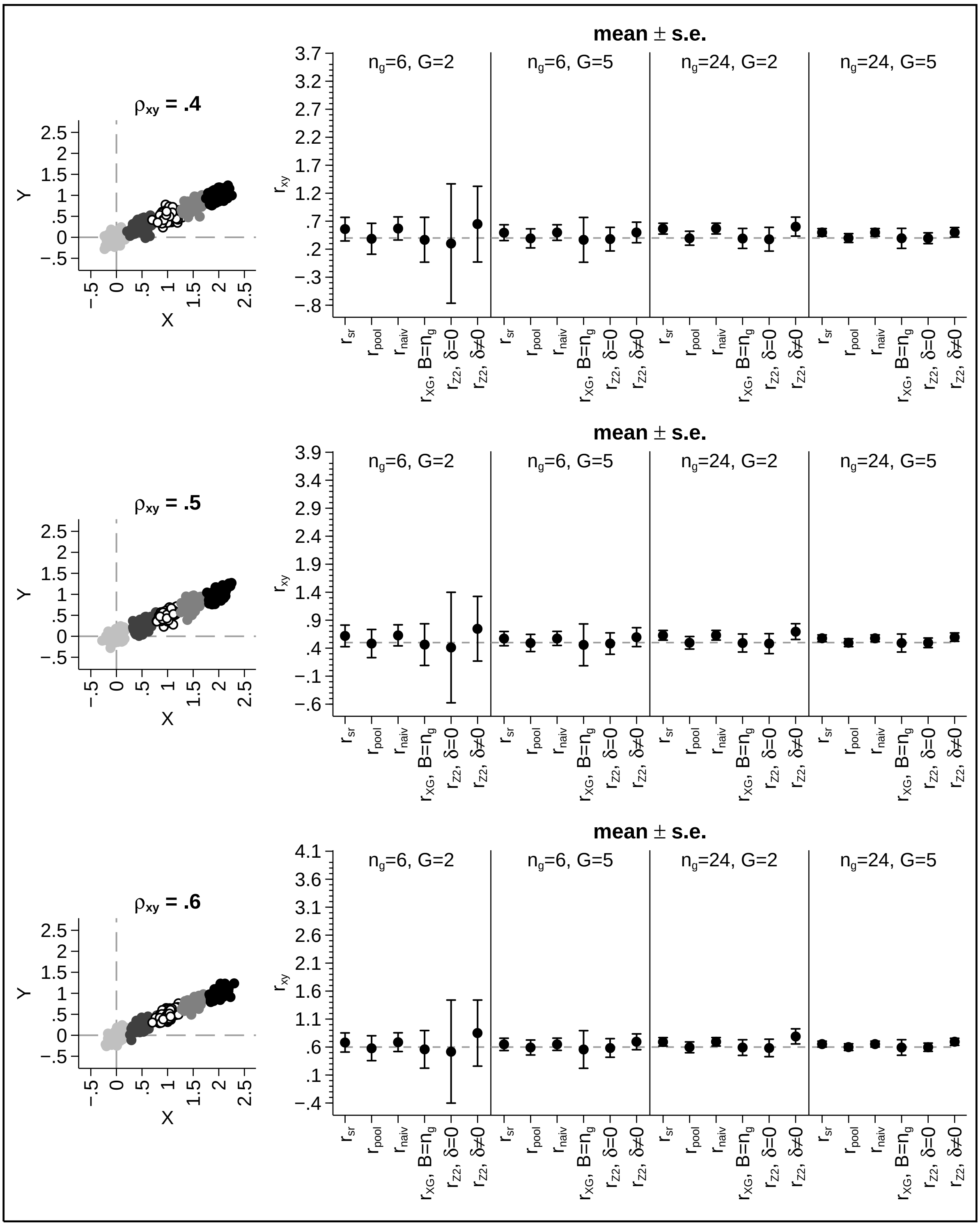

Figure 4.5: Mean (± s.e.) for the estimate of the correlation coefficient for Example 4

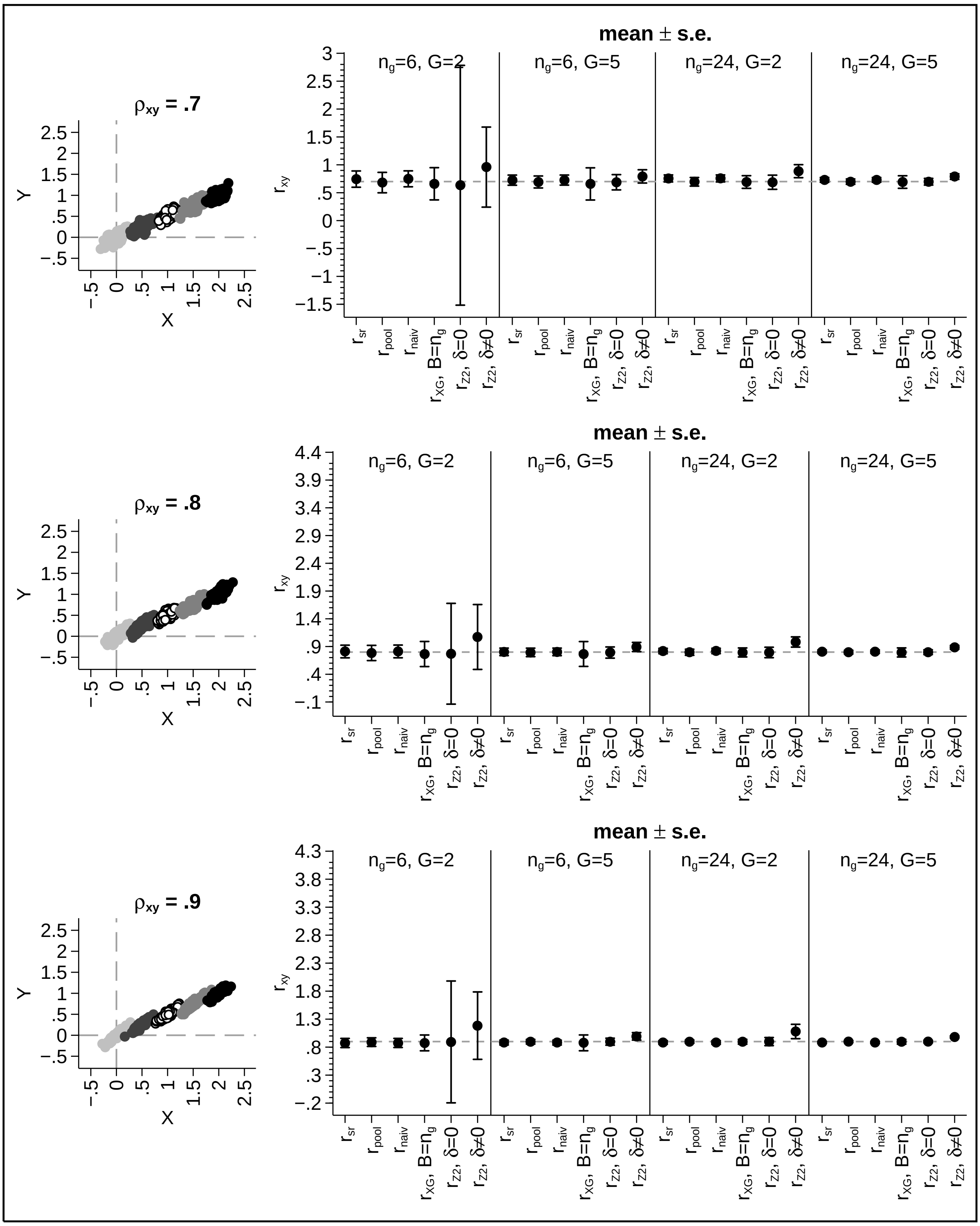

Figure 4.6: Mean (± s.e.) for the estimate of the correlation coefficient for Example 4
